# Origin and Length Distribution of Unidirectional Prokaryotic Overlapping Genes

**DOI:** 10.1534/g3.113.005652

**Published:** 2013-11-05

**Authors:** Miguel M. Fonseca, D. James Harris, David Posada

**Affiliations:** * Department of Biochemistry, Genetics and Immunology, University of Vigo, 36310 Vigo, Spain; † CIBIO, Research Center in Biodiversity and Genetic Resources, InBIO Laboratório Associado, 4485-661 Vairão, University of Porto, Portugal

**Keywords:** overlapping genes, prokaryotic genomes, unidirectional genes, overlap length distribution

## Abstract

Prokaryotic unidirectional overlapping genes can be originated by disrupting and replacing of the start or stop codon of one protein-coding gene with another start or stop codon within the adjacent gene. However, the probability of disruption and replacement of a start or stop codon may differ significantly depending on the number and redundancy of the start and stop codons sets. Here, we performed a simulation study of the formation of unidirectional overlapping genes using a simple model of nucleotide change and contrasted it with empirical data. Our results suggest that overlaps originated by an elongation of the 3′-end of the upstream gene are significantly more frequent than those originated by an elongation of the 5′-end of the downstream gene. According to this, we propose a model for the creation of unidirectional overlaps that is based on the disruption probabilities of start codon and stop codon sets and on the different probabilities of phase 1 and phase 2 overlaps. Additionally, our results suggest that phase 2 overlaps are formed at higher rates than phase 1 overlaps, given the same evolutionary time. Finally, we propose that there is no need to invoke selection to explain the prevalence of long phase 1 unidirectional overlaps. Rather, the overrepresentation of long phase 1 relative to long phase 2 overlaps might occur because it is highly probable that phase 2 overlaps are retained as short overlaps by chance. Such a pattern is stronger if selection against very long overlaps is included in the model. Our model as a whole is able to explain to a large extent the empirical length distribution of unidirectional overlaps in prokaryotic genomes.

Overlapping genes—genes that share nucleotides—were originally discovered in viruses (Barrell *et al.* 1976) but are also known to occur in prokaryotic, organelle, and eukaryotic genomes (Smith and Parkinson 1980; Montoya *et al.* 1983, Spencer *et al.* 1986). Overlapping genes regulate the expression of the genes involved through translational coupling in operons (Normark *et al.* 1983; Cooper *et al.* 1998, Zheng *et al.* 2002) and transcriptional regulation (Wagner *et al.* 2002), play an important role in genome compaction (Miyata and Yasunaga 1978; Sakharkar *et al.* 2005), and may be involved in the creation of new genes (Keese and Gibbs 1992). The evolution of overlapping coding regions is particularly constrained because they simultaneously code for two different genes and any mutation affects both of them (Krakauer 2000).

Overlapping genes can share a fraction of their sequence—*partial* or *terminal* overlap—or one of the genes can be completely nested within the other—*complete* or *internal* overlap. The overlaps are termed unidirectional or same-strand if the genes involved are transcribed in the same direction, whereas if the two are located on complementary strands the overlap is referred to as opposite-strand. In partial unidirectional overlaps, the 3′-end of the upstream gene (henceforth called gene 1) shares nucleotides with the 5′-end of the downstream gene (henceforth called gene 2) (head-to-tail, →→). In partial opposite-strand overlaps, both genes share a common termini and are classified as convergent (involving 3′-ends, tail-to-tail, →←) or divergent (involving 5′-ends, head-to-head, ←→).

In prokaryotic genomes, most overlapping genes are unidirectional, which probably reflects the common orientation of neighboring genes (Fukuda *et al.* 2003). This type of overlap can be generated if the loss of the start/stop codon in a gene (by deletion, point mutation, or frameshift) results in the elongation of that gene to the next in-frame start/stop codon located in the coding region of an adjacent gene. Regarding the overlapping genes originated by point mutations, no consensus exists about which gene is usually extended to form the overlap. A mutation can occur in the stop codon, extending the 3′-end of gene 1 (Fukuda *et al.* 1999, 2003; Rogozin *et al.* 2002; Sakharkar *et al.* 2005), or in the start codon, extending the 5′-end of gene 2 (Cock and Whitworth 2007, 2010; Sabath *et al.* 2008).

Importantly, unidirectional gene overlaps may occur in different translational phases, such as phase 0, phase 1, or phase 2, if the first codon position of gene 1 is the first, second, or third codon position of gene 2, respectively (Figure 1). Phase 0 overlaps can be considered just as alternative start sites of the same gene and are not considered here. In phase 1 and phase 2 overlaps, gene 1 and gene 2 are frameshifted one or two nucleotides, respectively. Moreover, overlaps can be classified as short if the stop codon of gene 1 and the start codon of the gene 2 share less than six nucleotides; otherwise, they are classified as long. Short overlaps are mainly found in phase 2, which is expected because in phase 1 the number of possible combinations given the start/stop codons sets is smaller because of the nature of the genetic code (three possible combinations for short phase 1 overlaps and eight combinations for short phase 2 overlaps). Furthermore, short phase 1 overlaps require ATT to be the start codon of gene 2, which is extremely rare, in combination with any of the stop codons (AT**T**AA, AT**T**AG, or AT**T**GA), whereas short phase 2 overlaps can choose different combinations of start and stop codons. For example, if the stop codon of gene 1 ends in A (TG**A** or TA**A**) or G (TA**G**), then in phase 1 this site can become the first codon position of the start codons **A**TG/**A**TT or **G**TG, respectively. Alternatively, in phase 2 short overlaps, the stop codon TGA of gene 1 can share its first two positions (**TG**A) with the last two positions of the common start codons (A**TG**, G**TG** or T**TG**) of gene 2 and create a four-nucleotide-long overlap—(A|G|T)TGA.

In long overlaps, phase 1 is the predominant phase (Fukuda *et al.* 2003) and, in recent years, different hypotheses have been put forward to explain this observation. Kingsford *et al.* (2007) showed that random 3′-end extensions of genes to the next occurring downstream stop codon could explain major patterns of the distribution of overlap lengths distribution in oppositely oriented genes (convergent overlaps). A similar effect was observed among unidirectional gene pairs (published in supporting information of Kingsford *et al.* 2007). Cock and Whitworth (2007) suggested that most overlaps originate through a 5′-end extension of gene 2 and that selection would favor long overlaps in phase 1 over phase 2 because phase 1 maximizes the mutational redundancy of the original gene (gene 1) and promotes sequence mutability of the newly extended region of gene 2. However, Sabath *et al.* (2008) proposed that compositional (the structure of the genetic code and amino-acid composition) rather than selective factors would be responsible for the predominance of long phase 1 overlaps. They observed that the empirical frequency of the stop codons did not differ significantly between phases, whereas start codons were more frequent in phase 1 overlaps, and suggested that the higher frequency of potential start codons in phase 1 would favor the formation of overlaps by 5′-end elongation of gene 2, increasing long phase 1 overlaps. Recently, Cock and Whitworth (2010) also suggested that most overlaps were created by the elongation of the 5′-end of gene 2, based on the distribution similarity between real overlaps and simulated overlaps of real nonoverlapping neighboring genes.

**Figure 1:**
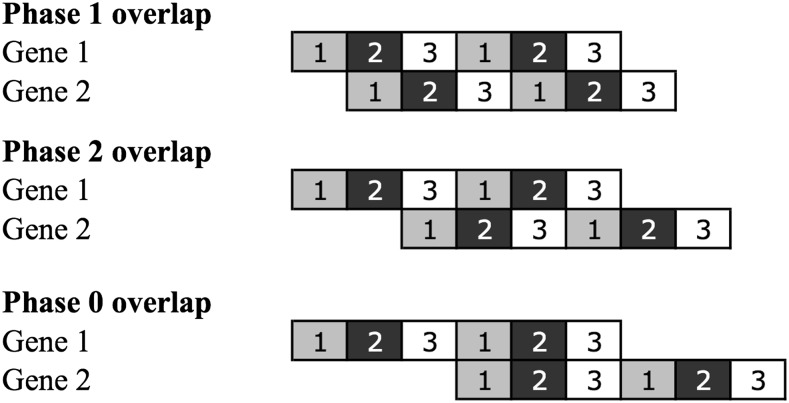
Schematic representation of the three possible phases of unidirectional overlapping genes. Numbers in boxes indicate codon position of each gene. Phase 0 overlaps can be considered just as alternative start sites of the same gene and were not considered in this study.

Contradicting these results, some empirical studies suggest that most overlaps are formed by the elongation of the 3′-end of gene 1 (Fukuda *et al.* 1999, 2003; Sakharkar *et al.* 2005). Importantly, all studies assumed that the disruption of start and stop codons is equally probable, although this seems unlikely given that start and stop codons differ in their robustness to mutations just because of the genetic code. In summary, a conclusive explanation for the distribution of unidirectional gene overlaps is still lacking. Here, we help solve this debate by answering three specific questions: (1) how will the robustness of start codon and stop codon sets influence the formation of overlapping genes?; (2) why most unidirectional overlapping genes occur in phase 2?; and (3) why is there a phase 1 predominance over phase 2 in long overlaps?

Using a simple model of random nucleotide change, we show that overlapping genes formed after the elongation of the 3′-end of gene 1 are twice as frequent as those originated by the elongation of the 5′-end of gene 2. We suggest that this is a consequence of the redundancy asymmetry between start codon and stop codon sets. Additionally, our results suggest that phase 2 overlaps are formed at higher rates than phase 1 overlaps, given the same evolutionary time. This could occur because shorter overlaps are more probable than longer overlaps (for both phases). However, only phase 2 overlaps can be short as a consequence of the genetic code itself. Thus, phase 2 overlaps have two more triplets that could work as alternative start/stop codons, making a phase 2 overlap more probable than a phase 1 overlap. Finally, we argue that there is no phase 1 selective preference in long overlaps; the bias favoring long phase 1 is, to a large extent, a simple consequence of the retention of most phase 2 overlaps as short overlaps and of the concentration of virtually all phase 1 overlaps as long overlaps.

## Materials and Methods

### Simulation of point mutations in two neighboring nonoverlapping genes

To test whether the formation of an overlapping region caused by elongation of gene 1 or gene 2 was equally frequent, we performed a computer simulation using a simple neutral model for the accumulation of nucleotide changes. We considered overlapping regions formed by point mutations and not by insertion/deletions based on the following observations to keep the simulations and the interpretations of the results simple: previous theoretical/*in silico* studies focused also on point mutations and nucleotide substitutions are much more common than indels (*i.e.*, the average ratio of nucleotide substitutions to indels for bacteria is 19.6) (Chen *et al.* 2009).

**Figure 2:**
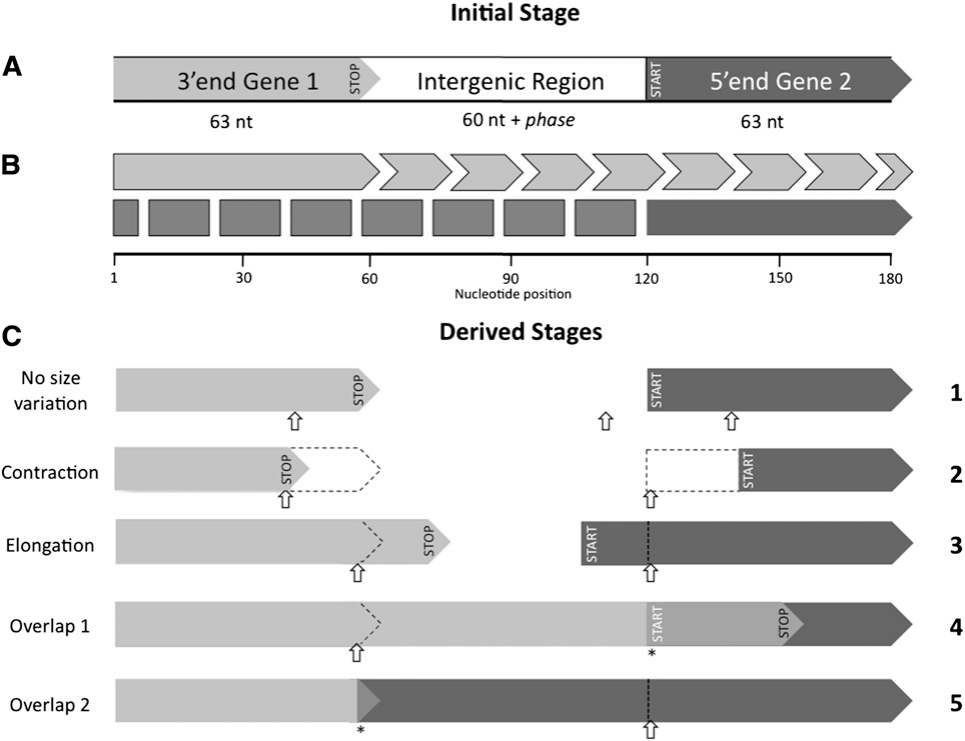
Scheme of the overlapping genes formation simulations (scenario 1). (A) In the initial stage ,we generated a sequence that contained the last 21 codons of gene 1 (including the stop codon) and the first 21 codons of gene 2 (including the start codon). These two partial gene sequences have the same direction of transcription and are separated from each other by 60 plus phase nucleotides. (B) Both genes have the same limits of maximum contraction (down to one codon) or elongation (up to the end of the sequence). Light gray arrows represent the limits of gene 1 size variation, and dark gray arrows and boxes represent gene 2. (C) Next, we made random changes (small open arrows) at the nucleotide level across the sequence until an overlapping region was created (*). No gene size variation occurred if the changes occurred in the intergenic region or if they caused a synonymous/nonsynonymous mutation within the coding region (C1). However, if the changes caused the disruption of the existing start/stop codons or the creation of upstream stop codon for gene 1, then genes may alter their length (C2, C3) and ultimately create an overlap (C4, long overlap; C5, short overlap). Gene size variation was allowed to occur even without the formation of an overlapping region.

We adopted the bacterial/archaeal genetic code (NCBI genetic code 11). We generated each initial sequence according to the different GC contents: 30%, 50%, and 70%. The empirical GC content among the bacterial genomes studied here varies between 13.5% and 74.9%. We take into account the effect of different codon usages by constraining our simulations to three distinct %GC scenarios. In a first set of simulations (scenario 1), we performed 100,000 replicates for each scenario. Each replicate consisted of a random 186-nt-long (plus one or two nucleotides, depending on the phase) sequence containing two neighboring and nonoverlapping protein-coding sequences (the 3′-end of gene 1, an intergenic region, and the 5′-end of gene 2) oriented in the same direction (Figure 2). Both gene ends started with the same length, 21 codons, and the intergenic distance was set to 60-nt-long (plus one or two nucleotides, depending on the phase). We defined these lengths to constrain the maximum overlapping region to 59-nt-long (Pallejà *et al.* 2008). Then, we chose a start codon of gene 2 either according to the empirical start codon usage in prokaryotic genomes (80% ATG, 17% GTG, and 3% TTG) or assuming equal usage (one-third each). The alternative start codon ATT was not included in the simulations because it is rarely observed in prokaryotic genomes (Cock and Whitworth 2010). The stop codon of gene 1 was chosen at random (one-third TAA, one-third TAG, and one-third TGA). At this initial stage, we did not accept stop codons within the coding sequence, except the predefined one at the 3′-end of gene 1. In a second step, we generated random nucleotide changes, one at a time, with uniform probability along the sequence until an overlapping region between genes 1 and 2 was formed. However, we only accepted these nucleotide changes if gene 1 still had a single stop codon in the sequence and if gene 2 still had a start codon and no in-frame stop codons (premature stop codons). Here, we assumed that the function of the gene was not altered by its elongation or contraction. The disruption of stop codons must necessarily extend the 3′-end of a gene until the next occurring downstream stop codon is found. However, the elongation of the 5′-end of a gene after start codon disruption might depend on the availability of other genomic features [*i.e.*, ribosome-binding sites (RBS)]. To keep the simulations simple, we did not include RBS. Previous studies suggest that a large number of prokaryotic genes initiate translation without possessing RBS (Skorski *et al.* 2006; Scharff *et al.* 2011).

The first in-frame stop codon for gene 1 defined the limit of its 3′-end. Gene 1 extended only with the disruption of the functional stop codon and if another stop codon was present downstream in the same frame (Figure 2, C3 and C4). The contraction of gene 1 occurred when a nonstop codon within this gene experienced a change that resulted in a premature stop codon (Figure 2, C2). Gene 2 changed its 5′-end position only when its start codon was disrupted. Then, we defined two possible scenarios: gene 2 contracted or elongated, depending on whether the next in-frame start codon occurred downstream (gene contraction) (Figure 2, C2) or upstream (gene elongation) (Figure 2, C3 and C5) of the original (and disrupted) start codon, respectively. However, we also included a third scenario in which potential start codons occurred downstream and upstream of the disrupted start codon at the same time. Which start codon should be used? To account for this, we performed the simulations alternatively under the following three scenarios regarding the disruption of the start codon of gene 2: “elongation first,” with preference for gene 2 elongation, *i.e.*, we first searched for a potential start codon in the upstream region of the disrupted start codon; “contraction first,” with preference for gene 2 contraction, *i.e.*, we first searched for a potential start codon in the downstream region of the disrupted start codon; and “both,” with gene 2 elongation/contraction being equally probable, *i.e.*, we randomly searched for a potential start codon with no preference for the downstream or upstream region of the disrupted start codon. The simulation ended when genes 1 and 2 overlapped. At the end of each replicate, we recorded the gene that “caused” the overlap and the length of the resultant overlap, so we ended up with overlap length distributions for the different scenarios and phases. Finally, for each run we also recorded the total number of mutations tried as a surrogate for evolutionary time. Because each replicate consisted of only one set of two adjacent genes, we could easily specify whether the overlap would finally occur in phase 1 or in phase 2, simply by setting the intergenic distance between gene 1 and gene 2 to 61 and 62 nucleotides for phase 1 and phase 2 simulations, respectively. However, in prokaryotic genomes, both phases are not equally frequent, with phase 2 overlaps being approximately 2.5-times more frequent than phase 1 overlaps (Fukuda *et al.* 2003; Sabath *et al.* 2008; Cock and Whitworth 2010). To make meaningful quantitative comparisons between simulated and real phase 1/phase 2 frequencies, we weighted (*a posteriori*) the representativeness of each simulated phase in the overall overlap length distribution. In the simulations the sequences evolved until an overlap was formed, but this time was longer for phase 1 than for phase 2 overlaps. However, in real data phase 1 overlaps necessarily resulted from the same evolutionary time than phase 2 overlaps. Therefore, we weighted the phase 1/phase 2 frequencies obtained in the simulation with the number of mutations, which is a surrogate for the average time needed for phase 1/phase 2 overlaps to occur.

We also performed two more simulations (scenarios 2 and 3) to test the effect of selection against very long overlaps (>60 bp). In these scenarios, gene size and intergenic distance were retrieved from an empirical distribution of prokaryotic genomes (Figure S1, Figure S2, and Figure S3) and no *a posteriori* weighting scheme was applied. For scenario 2 the overlap length had no restriction (apart from the full length of the overlapped gene), whereas in scenario 3 only overlaps shorter than 60 bp were accepted. In both simulation sets, gene sizes and intergenic distances were limited (50–1000 codons for gene size; 0–99 plus phase for intergenic distance) to make computations feasible.

### Length distribution of real unidirectional prokaryotic overlapping genes

To compare the results of our simulations with real data, we retrieved nucleotide sequences from unidirectional overlapping gene pairs (OGPs) from 2151 prokaryotic genomes available in NCBI (until 20 September 2012; plasmid and phage sequences were excluded). To avoid counting near-identical OGPs, we grouped them according to sequence similarity using pairwise *blastx* searches. We clustered different OGPs if their amino acid sequence identity was ≥70% and if the open reading frame (ORF) coverage was ≥80%. We grouped the initial 1,076,734 OGPs into 84,258 clusters containing at least two OGPs. The remaining 247,532 OGPs were considered unique. From each cluster, we selected one OGP for each unique overlap length. Overlaps larger than 59 bp were not analyzed because these could contain a large number of misannotations (Pallejà *et al.* 2008). The final nonredundant dataset of unique OGPs consisted of 400,363 OGPs, from which we constructed the distribution of overlap length. We also calculated the overlap length distributions for different taxa in different domain and phyla and used them to test whether the overall frequency of short and long phase 1 and phase 2 overlaps was significantly different across groups. Finally, for each genome and for each phase, we computed the proportion of real overlapping genes relative to the potential ones, *i.e.*, unidirectional but nonoverlapping genes, to test if the degree of success of overlap formation differed between phases.

## Results

### Simulation of point mutations in two neighboring nonoverlapping genes

**Figure 3:**
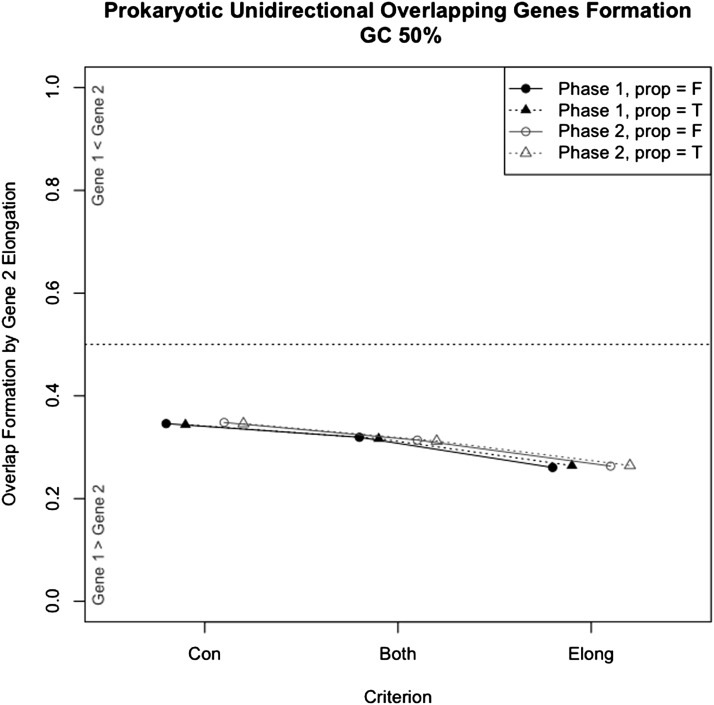
Proportion of overlaps caused by the elongation of gene 2 (downstream gene). The proportion of overlapping genes formed after the disruption of the start codon and elongation of gene 2 was significantly lower than the proportion formed by the elongation of gene 1 (*P* ≤ 0.001) for all simulations (scenario 1). Data shown correspond to the 50% GC content scenario, but the results with 30% and 70% GC content were similar (Supporting Information). The simulations were performed separately with three criteria: preference for gene 2 contraction (criterion “Con”); gene 2 elongation/contraction equally probable (criterion “Both”); and preference for gene 2 elongation (criterion “Elong”). Prop = F, start codons were chosen at random. Prop = T, start codons were chosen according to empirical codon usage in prokaryotic genomes (80% ATG, 17% GTG, and 3% TTG). Each simulation was replicated 10^6^ times.

**Figure 4:**
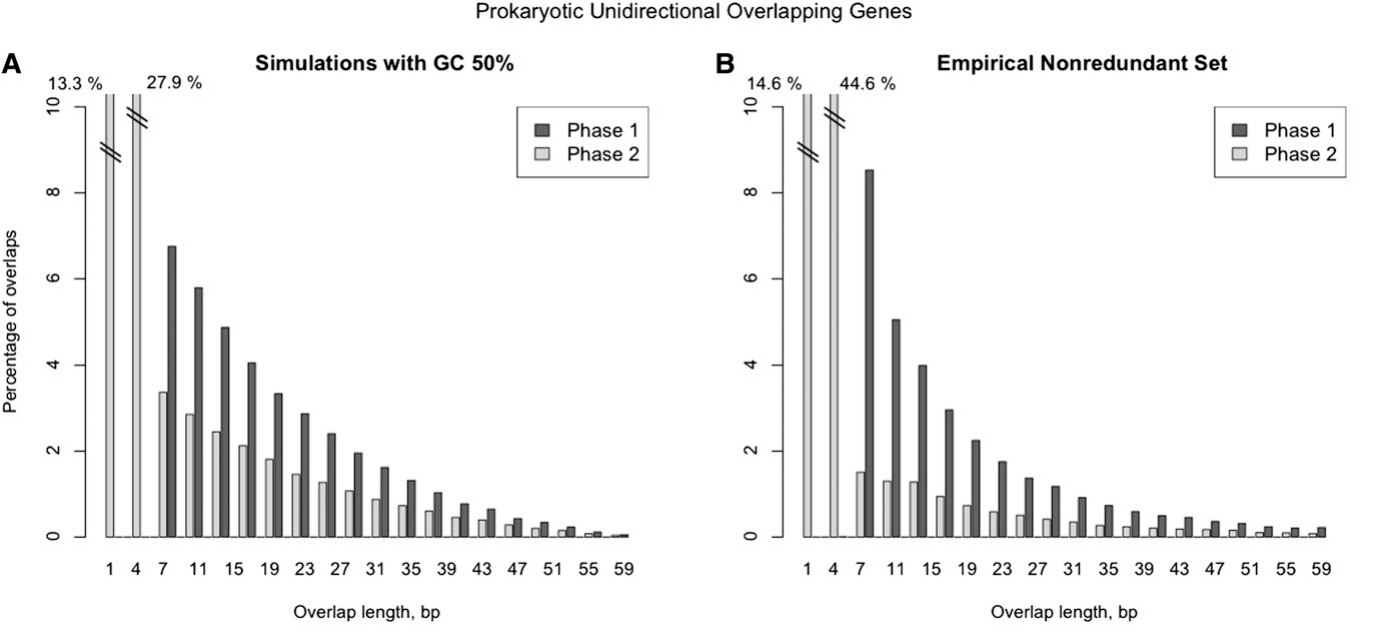
Comparison of the unidirectional overlapping gene length distributions between simulated (scenario 1) and empirical data. (A) Hypothetical prokaryotic overlap lengths of unidirectional adjacent genes, calculated from simulated dataset (simulated parameters: GC content = 50%; criterion = Both; proportions of start codons = TRUE). Frequencies of overlaps in both phases were weighted according to the mutation rate between phase 1 and phase 2 simulations (rate = 1.59; see Table 1). Frequency proportions used were as follows: phase 1 = 38.6%; and phase 2 = 61.4%. (B) Distribution of empirical prokaryotic overlap lengths of unidirectional adjacent genes calculated from a nonredundant set of 400,363 overlapping gene pairs (phase 1 = 31.6%; phase 2 = 68.4%).

**Figure 5:**
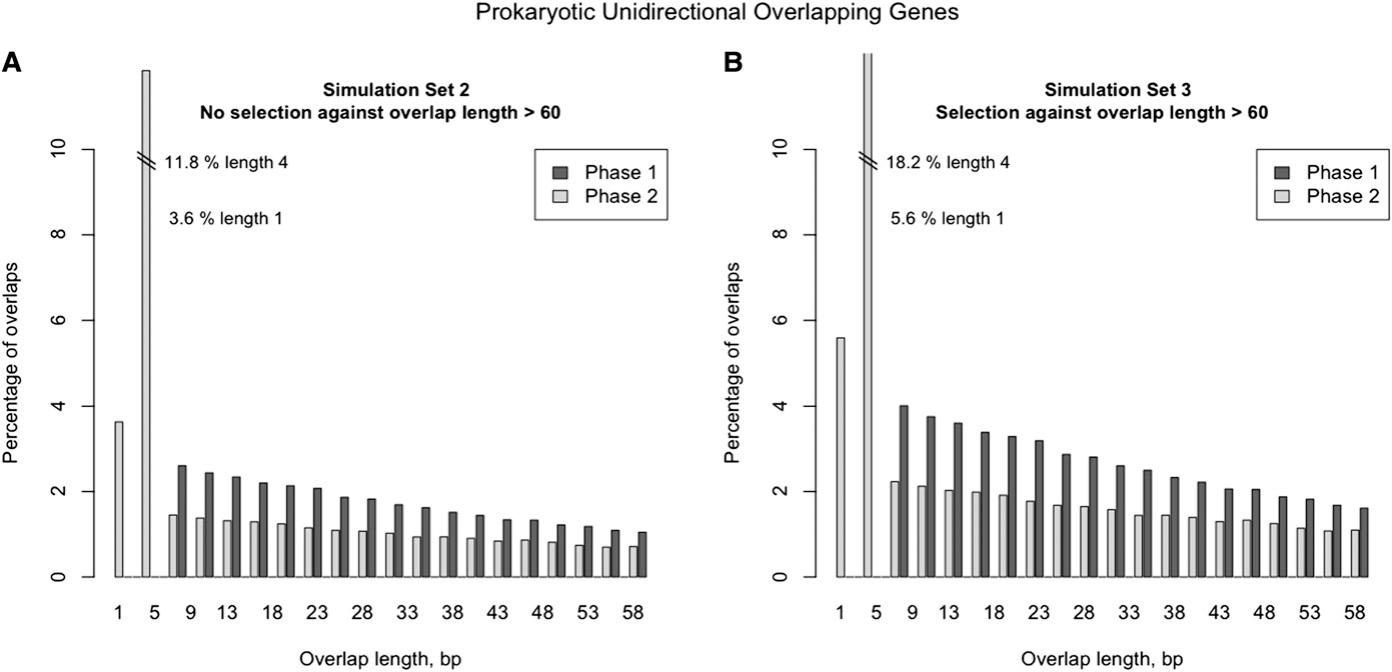
Comparison of the unidirectional overlapping gene length distributions between simulated scenarios (scenario 2 *vs.* scenario 3) testing the effect of selection against very long overlaps (overlaps >60 bp). These simulations differ from the ones presented in Figure 4A. Gene sizes and intergenic distances were retrieved from the empirical distribution of prokaryotic genomes and no *a posteriori* weighting scheme was applied to the representativeness of phase 1 or phase 2. For practical reasons, gene sizes and intergenic distances were limited (50–1000 codons for gene size; 0–99 plus phase for intergenic distance). (A) Simulation scenario 2. Hypothetical prokaryotic overlap lengths of unidirectional adjacent genes calculated from simulated dataset (simulated parameters: GC content = 50%; criterion = Both; proportions of start codons = TRUE). No selection against overlap length >60 bp was included. Bar plot is limited to show only overlap length <60 bp. (B) Simulation scenario 3. Hypothetical prokaryotic overlap lengths of unidirectional adjacent genes calculated from simulated dataset (simulated parameters: GC content = 50%; criterion = Both; proportions of start codons = TRUE). Selection against overlap length >60 bp was included.

In simulation scenario 1, overlapping regions originated by the elongation of the 3′-end of gene 1 were 1.9-times to 2.8-times more frequent than those originated by the 5′ elongation of gene 2 (*P* ≤ 0.001) (Figure 3, Figure S5, and Figure S6). Phase 1 simulations required approximately 1.5-times more mutations than phase 2 simulations to form overlaps (Table 1). All simulated sets showed the same overall overlap length distribution (Figure 4A, Figure 5, A and B, Figure S7, Figure S8, Figure S9, Figure S10, Figure S11, Figure S12, Figure S13, Figure S14, and Figure S15), with the majority of phase 2 overlaps being short. The most frequent overlap length was 4 nt, followed by 1-nt overlaps. All phase 1 overlaps were long overlaps, and long overlaps were more frequent in phase 1 than in phase 2. When scenarios 2 and 3 were compared, we observed that scenario 3, which includes selection against very long overlaps, resulted in an overlap length distribution most similar to the empirical distribution (Figure 5). Variation in GC content equally affected scenarios 2 and 3: low %GC resulted in shorter overlaps on average (Figure S7, Figure S8, Figure S9, Figure S10, Figure S11, Figure S12, Figure S13, Figure S14, and Figure S15). In addition, short overlaps were more frequent in genomes with low GC content (scenario 2, no limit for overlap length) (Figure S10, Figure S11, and Figure S12).

**Table 1:** Average number of mutations per replicate of phase 1 *vs.* phase 2 scenarios (simulation set 1)

GC Content, %	Simulation Criterion*^a^*	Start Codon Proportions, T/F	Phase 1 Mutations, Average Per Replicate	Phase 2 Mutations, Average Per Replicate	Ratio of Phase 1 to Phase 2
30	Contraction	F	8792	5642	1.56
		T	8729	5677	1.54
	Both	F	8520	5345	1.59
		T	8418	5339	1.58
	Elongation	F	5051	3227	1.57
		T	5045	3230	1.56
50	Contraction	F	9115	5812	1.57
		T	9043	5828	1.55
	Both	F	8863	5553	1.60
		T	8773	5507	1.59
	Elongation	F	5168	3277	1.58
		T	5144	3274	1.57
70	Contraction	F	9379	5987	1.57
		T	9303	5927	1.57
	Both	F	9068	5713	1.59
		T	9042	5689	1.59
	Elongation	F	5321	3361	1.58
		T	5315	3344	1.59

The 18 possible scenarios (combining simulation criterion with GC content and start codon proportions) are shown (for a full description of the parameters used, see *Material and Methods*). The average of mutations was calculated using all 100,000 replicates of each scenario. F, false; T, true.

a Criterion used to search for potential start codons when the functional start codon is disrupted. Preference for gene 2 contraction (“Contraction”), preference for gene 2 elongation (“Elongation”), no preference, and both scenarios equally probable (“Both”).

### Length distribution of real unidirectional prokaryotic overlapping genes

**Figure 6:**
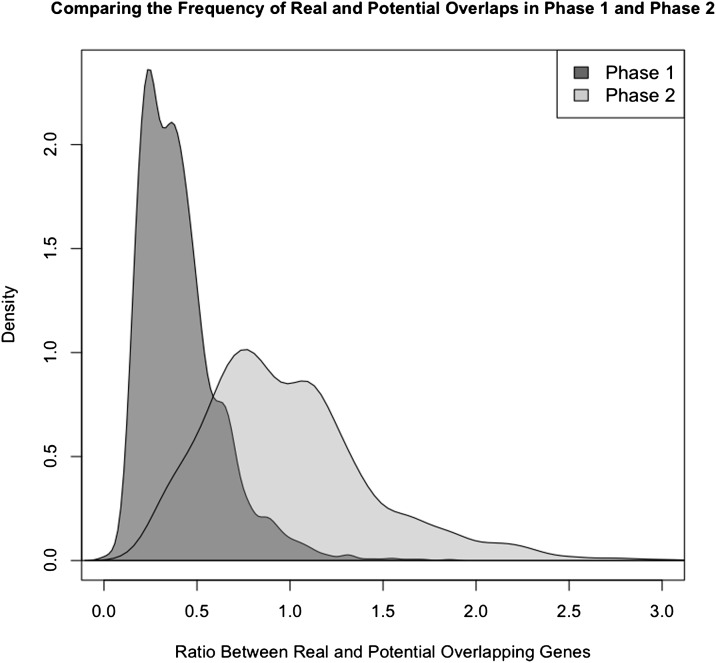
Density plot of the ratio between real and potential prokaryotic unidirectional overlapping genes. Data were computed for each genome and for each overlapping phase. The relative degree of success of phase 2 overlaps is significantly higher than the degree of success of phase 1 overlaps (Kolmogorov-Smirnov test, one-tailed test, *P* ≤ 0.001).

The empirical nonredundant set of prokaryotic overlap length distribution was very similar to the simulated distribution described. Thus, phase 2 represented more than 68% of all overlaps (Figure 4B). Most phase 2 overlaps were short (87% of all phase 2 overlaps and 59% of all overlaps), whereas phase 1 overlaps were essentially long overlaps. Long overlaps were significantly more frequent in phase 1 than in phase 2 (Table S1). The most frequent overlap length was 4-nt-long (short phase 2 overlap; representing approximately 45% of all overlaps), and the second most frequent overlap length was 1-nt-long (also short phase 2 overlap; representing approximately 15% of all overlaps). A similar trend was found within all taxonomic groups analyzed (Table S1). The ratio between observed and potential overlaps was significantly higher in phase 2 than in phase 1 overlaps (Figure 6) (Kolmogorov-Smirnov test, one-tailed test, *P* ≤ 0.001).

The empirical data show a negative correlation between GC content and frequency of long phase 1 and, to a lesser extent, phase 2 overlaps (long phase 1, *r^2^* = 0.72, *P* < 0.001; long phase 2, *r^2^* = 0.06, *P* < 0.001) (Figure S19) and a positive correlation between the frequency of short phase 2 overlaps and GC content (*r^2^* = 0.69, *P* < 0.001) (Figure S19).

The empirical GC content was negatively correlated with the frequency of long phase 1 (*r^2^* = 0.72, *P* < 0.001) and long phase 2 (*r^2^* = 0.06, *P* < 0.001) overlaps, but was positively correlated with the frequency of short phase 2 overlaps (*r^2^* = 0.69, *P* < 0.001) (Figure S19).

## Discussion

### *In silico* patterns of overlapping genes formation

We have shown that unidirectional gene overlaps caused by the elongation of the upstream gene (gene 1) are more frequent than those caused by elongation of the downstream gene (gene 2) when stochastic nucleotide changes are simulated in a sequence containing two adjacent and nonoverlapping protein-coding genes. We believe that this pattern of formation of overlapping genes is attributable, in part, to the different robustness of the start codon and stop codon sets. Some nucleotide changes in start/stop codons are functionally redundant, *i.e.*, changes that convert a start codon into another start codon, or a stop codon into another stop codon. Under the same mutational pressures, the prokaryotic stop codon set is less redundant/robust than the start codon set, because only 4 out of 27 nucleotide changes will be functionally redundant. For the start codon set, 6 out of 27 possible nucleotide changes are functionally redundant. Thus, under a stochastic model of nucleotide change, the disruption probability for the start codon set is 0.78, whereas for the stop codon set it is 0.85. If stop codons are more frequently disrupted, then the 3′-end of protein-coding genes will also vary more frequently, and its elongation could, eventually, originate more gene overlaps than the elongation of the 5′-end.

Interestingly, several empirical studies have also pointed out that overlapping regions originated by the 3′-end elongation of gene 1 are more frequent (Fukuda *et al.* 1999, 2003; Sakharkar *et al.* 2005). However, redundancy asymmetry was not invoked to explain it. Instead, it was suggested that 3′-end elongation originates more overlapping genes because selective constraints are stronger at the 5′-end than at the 3′-end of protein-coding genes. The logic is that because the 5′-end together with its upstream region incorporates essential structures such as promoters, then it should be more constrained in size variation and consequently would originate less gene overlaps (Rogozin *et al.* 2002; Fukuda *et al.* 2003; Sakharkar *et al.* 2005). The two hypotheses, the redundancy asymmetry of start/stop codons and the different selective constraints between the 5′-end and 3′-end, are not mutually exclusive; therefore, both should be considered in future studies.

On the contrary, *in silico* and theoretical studies have proposed that most unidirectional overlaps are formed with the elongation of the 5′-end of gene 2 (Cock and Whitworth 2007, 2010; Sabath *et al.* 2008). However, these studies focused on features that could influence the patterns of overlapping genes formation, but, importantly, given that the disruption of a start/stop codon already occurred. Our study suggests that taking into account the redundancy asymmetry between start/stop codon sets is key because both codon sets are not equiprobably disrupted, as we have shown. Additionally, we allowed elongation of gene 1 or gene 2 to freely occur in each simulation, which we think is more realistic than considering both elongations to be (almost) mutually exclusive (Cock and Whitworth 2010).

Other factors or features could also affect the patterns of the formation of overlapping genes, such as variable mutation rates, Shine-Dalgarno translation initiation sites, and different selective constraints between 5′-end and 3′-end elongations or between 5′-UTRs and 3′-UTRs. However, the inclusion of these additional factors would complicate the interpretation of the observed patterns. Our strategy here was to simulate simple scenarios to understand the main features of the overlapping process; in fact, our results were in agreement with those of previous empirical studies (Rogozin *et al.* 2002; Fukuda *et al.* 2003; Sakharkar *et al.* 2005).

### Overlap length distribution in unidirectional overlapping genes

The resulting simulated and empirical overlap length distributions share four relevant features: phase 2 overlaps are more frequent than those in phase 1; the most frequent type of overlaps is short phase 2; the frequency of long overlaps decreases with the length of the overlap; and in long overlaps, those in phase 1 are more frequent.

Previous studies have proposed that the evolution of overlapping genes may be related to the evolutionary time-scale, suggesting that they occur at a constant rate across species (Fukuda *et al.* 1999, 2003; Johnson and Chisholm 2004) (Supporting Information). However, these studies did not distinguish between the evolutionary rates of phase 1 and phase 2 overlaps, which in turn could affect their proportion within a given genome. In set 1 simulations, the average number of mutations in phase 2 overlaps will be proportional to the evolutionary time, suggesting that in prokaryotic genomes phase 2 overlaps should form at a higher rate than phase 1 overlaps. We should also consider that phase 2 overlaps can be short, but all phase 1 overlaps have to be long if the (rare) start codon ATT is not used, as in our study. Thus, given the same potential elongation length, phase 2 overlaps have more length categories in which potential overlaps can occur (one-nucleotide and four-nucleotide overlaps). Ultimately, this may explain the higher formation rate for phase 2 overlaps. The fact that the proportion between real and potential overlaps is significantly higher in phase 2 supports this idea.

Although translational coupling provides a biological justification for the higher frequency of short overlaps (Lillo and Krakauer 2007; Cock and Whitworth 2010), our simulations suggest that these overlaps could be partially obtained without including any selective advantage (Lillo and Krakauer 2007). In fact, just looking at the genetic code we can realize that a short overlap is highly likely to occur after the disruption of a start or stop codon. For example, to form a 4-nt overlap with the elongation of the 3′-end of a gene, we only need to have an adenine after the start codon (ATG**A**, GTG**A,** or TTG**A**) of gene 2, forming a TGA stop codon. Likewise, after the elongation of the 5′-end of gene 2, we only need to have an adenine just before the stop codon **A**TGA to form an ATG start codon and therefore a 4-nt overlap. In previous simulation studies, the higher frequency of short phase 2 overlaps was also recovered without providing any selective advantage to short phase 2 overlaps (Kingsford *et al.* 2007; Cock and Whitworth 2010). Kingsford *et al.* (2007) suggested that the overlap length distribution in unidirectional overlaps was significantly determined by the expected location of the stop codons for gene 1 within gene 2. On the contrary, Cock and Whitworth (2010) indicated that the overlap length distribution was instead significantly determined by the expected location of the start codons for gene 2 within gene 1. Importantly, we highlight the fact that in these studies only one scenario—elongation of gene 1 or elongation of gene 2—was invoked to explain the overlap length distribution. Here, we integrated both scenarios and recovered an overlap length distribution in agreement with that of the empirical data. Our results suggest that both the expected location of potential stop codons for gene 1 within gene 2 and the expected location of potential start codons for gene 2 within gene 1, together with the redundancy asymmetry between start and stop codons sets, can explain the higher frequency of short phase 2 overlaps. However, translational coupling, as a biological justification for the higher frequency of short overlaps (Kingsford *et al.* 2007; Cock and Whitworth 2010), could also be relevant in shaping the distribution of overlap length given the differences we found between simulated and empirical distributions.

As in prokaryotic genomes, in our simulations the frequency of long overlaps decreased with the length of the overlap. However a lower decay rate with increasing overlap length resulted in “less shorter” overlaps and “more longer” overlaps for the simulation set with no selection against overlap length larger than 60 bp. These results suggest that selection against very long overlaps plays an important role in shaping real overlap length distribution, as previously suggested (Kingsford *et al.* 2007; Cock and Whitworth 2010). Nevertheless, our results also indicate that major patterns in the overlapping length distribution (short phase 2 as the most frequent; long phase 1 overlaps more frequent than long phase 2) and a decay rate can be partially explained by the probability of finding frameshifted start and stop codons across a protein-coding gene and by taking into account start/stop codon redundancy.

Finally, both the simulated and the empirical overlap length distributions had a bias favoring long phase 1 over long phase 2 overlaps. If both phases have the same selective constraints (Krakauer 2000; Lillo and Krakauer 2007), then why are long overlaps mainly in phase 1? Existing hypotheses involve selective factors (Cock and Whitworth 2007), compositional factors (Sabath *et al.* 2008), the genetic code itself, and intergenetic distances coupled with selection against longer overlaps (Kingsford *et al.* 2007; Cock and Whitworth 2010). All these studies compared intrinsic features between long phase 1 and phase 2 overlaps that could explain the higher frequency of long phase 1 overlaps. We hypothesize that short overlaps will have priority over long overlaps because the overlap limit will be defined by the first in-frame start/stop codon within the neighboring gene. In other words, an overlap will only be long if it cannot be short. In our simulations, most phase 2 overlaps were short, whereas all phase 1 overlaps were long. Very likely, phase 2 overlaps were “retained” as short and, for this reason, long phase 2 overlaps were underrepresented. It should be highlighted that short phase 2 retention is stronger if overlap length is limited up to 60 bp, suggesting again that selection against longer overlaps also plays a role in the overlapping genes length distribution.

### Genomic GC content and overlap length distribution

The formation of overlapping genes depends on the existence of frameshifted start/stop codons, and the frequency of frameshifted start/stop codons decreases with increasing genomic GC content (Sabath *et al.* 2008). Lower frequencies of frameshifted start/stop codons favor the formation of longer overlapping genes. Thus, under neutrality, genomes with low GC content, which have lower frequencies of frameshifted start/stop codons, should have longer overlapping genes on average, and the proportions of long overlaps should increase over short overlaps. We did observe such a pattern in the simulations (scenario 2). However, the empirical data show a negative correlation between GC content and frequency of long phase 1 and, to a lesser extent, of phase 2 overlaps, which contradicts neutral expectations. Purifying selection against long overlaps could explain this negative correlation between long overlap frequency and GC content (Kingsford *et al.* 2007; Sabath *et al.* 2008; Cock and Whitworth 2010). Longer overlapping gene regions are more probable in genomes with higher GC content; therefore, stronger purifying selection is expected against these overlaps, leading to lower frequencies of long overlaps.

Likewise, in the absence of selection, short overlaps should be more frequent in low GC content genomes. However, empirical data show a positive correlation between the frequency of short phase 2 overlaps and GC content. Positive selection for translational coupling of pairs of genes may explain this observation: in genomes with high GC content, in which selection acts against longer overlaps, short overlaps are formed to maintain the translational coupling of the gene pairs involved.

## Conclusions

In general, our integrated approach is concordant with previous studies in showing that the length distribution of unidirectional overlaps can be, in part, influenced by the genetic code (Cock and Whitworth 2010) and by the expected location of potential start (Lillo and Krakauer 2007; Sabath *et al.* 2008; Cock and Whitworth 2010) and stop codons (Lillo and Krakauer 2007; Kingsford *et al.* 2007). Additionally, we advocate that it is important to take into account the asymmetric redundancy of start/stop codon sets and also to consider the two possible scenarios, elongation of gene 1 or gene 2. Finally, we suggest that phase 2 overlaps are more frequent and occur at higher evolutionary rates than phase 1 overlaps because only phase 2 overlaps can be short, which in turn is the most probable category of overlap.

To better understand the formation of overlapping genes, one could extend these simulations incorporating selective pressures in the novel overlapping regions (Sabath and Graur 2010) or include more complex structures, such as Shine-Dalgarno translation initiation sites. Further simulated and empirical studies are needed to decipher the contribution of overlapping gene death or overlap length contraction in the distribution of prokaryotic or viral overlapping genes (for eukaryotic genomes, see Makalowska *et al.* 2007). We hope our results are useful to research related to the origin and evolution of internal overlaps formed by overprinting (Sabath *et al.* 2012), especially if redundancy asymmetries between start and stop are taken into account.

## Supplementary Material

Supporting Information
