# Supplementary material for: Origin and Length Distribution of Unidirectional Prokaryotic Overlapping Genes
Source: G3 (Bethesda). 2013 Nov 5;4(1):19–27. doi: 10.1534/g3.113.005652 (PMC3887535; doi:10.1534/g3.113.005652)
Supplement: Supporting Information [file supp_4_1_19__index.html]

Origin and Length Distribution of Unidirectional Prokaryotic Overlapping Genes — Supporting Information 

# Origin and Length Distribution of Unidirectional Prokaryotic Overlapping Genes

## Supporting Information for Fonseca, Harris, and Posada, 2014

**Files in this Data Supplement:**

- Supporting Information - Figures S1-S19 and Table S1 (PDF, 2 MB)
- Figure S1 - Cumulative frequency of the distance (in bp) between neighboring unidirectional non-overlapping genes. (PDF, 345 KB)
- Figure S2 - Prokaryotic Gene Size Empirical Distribution (PDF, 309 KB)
- Figure S3 - Intergenic Distances used in phase 1 and phase 2 simulations (scenarios 2 and 3). (PDF, 316 KB)
- Figure S4 - Gene sizes used in the simulations (scenarios 2 and 3). (PDF, 325 KB)
- Figure S5 - Proportion of overlaps caused by the elongation of gene 2. Data shown corresponds to the 30% GC content scenario. (PDF, 470 KB)
- Figure S6 - Proportion of overlaps caused by the elongation of gene 2. Data shown corresponds to the 70% GC content scenario. (PDF, 470 KB)
- Figure S7 - Hypothetical prokaryotic overlap lengths of unidirectional adjacent genes, calculated from simulated dataset (scenario 1). Parameters: GC content = 30% (PDF, 614 KB)
- Figure S8 - Hypothetical prokaryotic overlap lengths of unidirectional adjacent genes, calculated from simulated dataset (scenario 1). Parameters: GC content = 50% (PDF, 614 KB)
- Figure S9 - Hypothetical prokaryotic overlap lengths of unidirectional adjacent genes, calculated from simulated dataset (scenario 1). Parameters: GC content = 70% (PDF, 615 KB)
- Figure S10 - Hypothetical prokaryotic overlap lengths of unidirectional adjacent genes, calculated from simulated dataset (scenario 2). Parameters: GC content = 30% (PDF, 501 KB)
- Figure S11 - Hypothetical prokaryotic overlap lengths of unidirectional adjacent genes, calculated from simulated dataset (scenario 2). Parameters: GC content = 50% (PDF, 500 KB)
- Figure S12 - Hypothetical prokaryotic overlap lengths of unidirectional adjacent genes, calculated from simulated dataset (scenario 2). Parameters: GC content = 70% (PDF, 499 KB)
- Figure S13 - Hypothetical prokaryotic overlap lengths of unidirectional adjacent genes, calculated from simulated dataset (scenario 3). Parameters: GC content = 30% (PDF, 500 KB)
- Figure S14 - Hypothetical prokaryotic overlap lengths of unidirectional adjacent genes, calculated from simulated dataset (scenario 3). Parameters: GC content = 50% (PDF, 500 KB)
- Figure S15 - Hypothetical prokaryotic overlap lengths of unidirectional adjacent genes, calculated from simulated dataset (scenario 3). Parameters: GC content = 70% (PDF, 500 KB)
- Figure S16 - Potential overlapping phase between adjacent non-overlapping gene pairs. (PDF, 310 KB)
- Figure S17 - Distribution of the skew values for the pairwise differences between potential phase 1 and potential phase 2 overlaps. (PDF, 429 KB)
- Figure S18 - (A) Relationship between genome length and number of unidirectional overlapping genes pairs. (B) Relationship between total number of unidirectional pairs of open reading frames (ORF) and the number of unidirectional overlapping genes pairs. (PDF, 498 KB)
- Figure S19 - Relative frequency of overlapping genes in 1453 prokaryotic genomes plotted against genomic GC content. (PDF, 577 KB)
- Table S1 - Proportions of prokaryotic unidirectional overlapping genes in phase 1 and phase 2. (PDF, 417 KB)
