# Supplementary material for: Origin and Length Distribution of Unidirectional Prokaryotic Overlapping Genes
Source: G3 (Bethesda). 2013 Nov 5;4(1):19–27. doi: 10.1534/g3.113.005652 (PMC3887535; doi:10.1534/g3.113.005652)
Supplement: Supporting Information [file supp_g3.113.005652_005652SI.pdf]

G3 · Genes | Genomes | Genetics

Origin and Length Distribution of Unidirectional Prokaryotic Overlapping Genes

Miguel M. Fonseca*,[1], §, D. James Harris§, David Posada*

* Department of Biochemistry, Genetics and Immunology, University of Vigo, 36310 Vigo, Spain

§ CIBIO, Research Center in Biodiversity and Genetic Resources, InBIO Laboratório Associado, 4485-661 Vairão, University of Porto, Portugal

[1]Corresponding author: University of Vigo, Department of Biochemistry, Genetics and Immunology, 36310 Vigo, Spain. Email: mig.m.fonseca@gmail.com

DOI: 10.1534/g3.113.005652

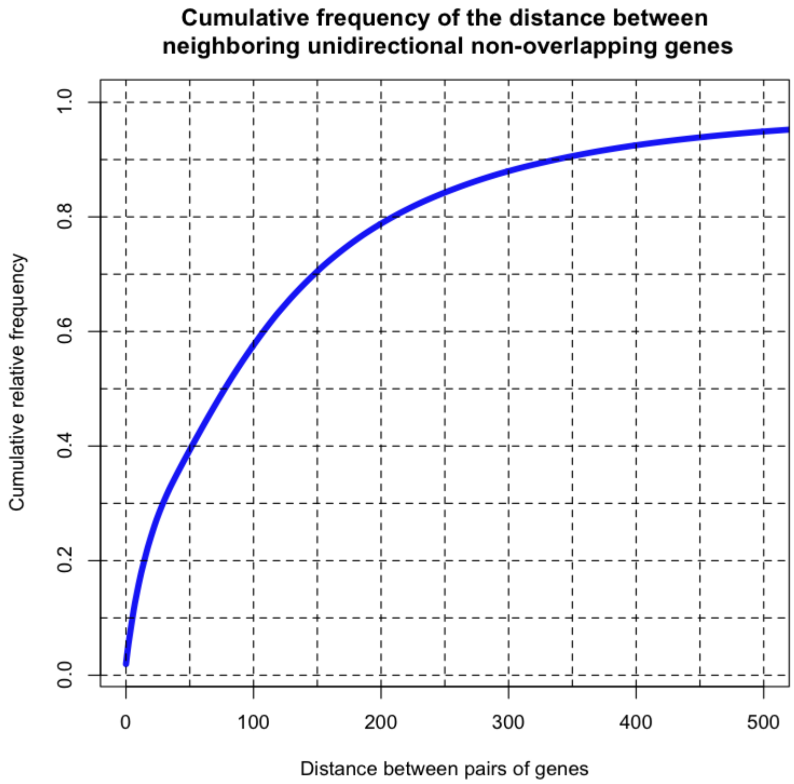

Figure S1 Cumulative frequency of the distance (in bp) between neighboring unidirectional non-overlapping genes. The figure shows intergenic distances up to 500 bp. The intergenic distances used in the simulations were retrieved from this empirical distribution. We have limited the distance up to 100 bp + phase for practical reasons. This interval [0-99 + phase] bp includes almost 60% of all empirical intergenic distances found between adjacent unidirectional genes in prokaryotic genomes.

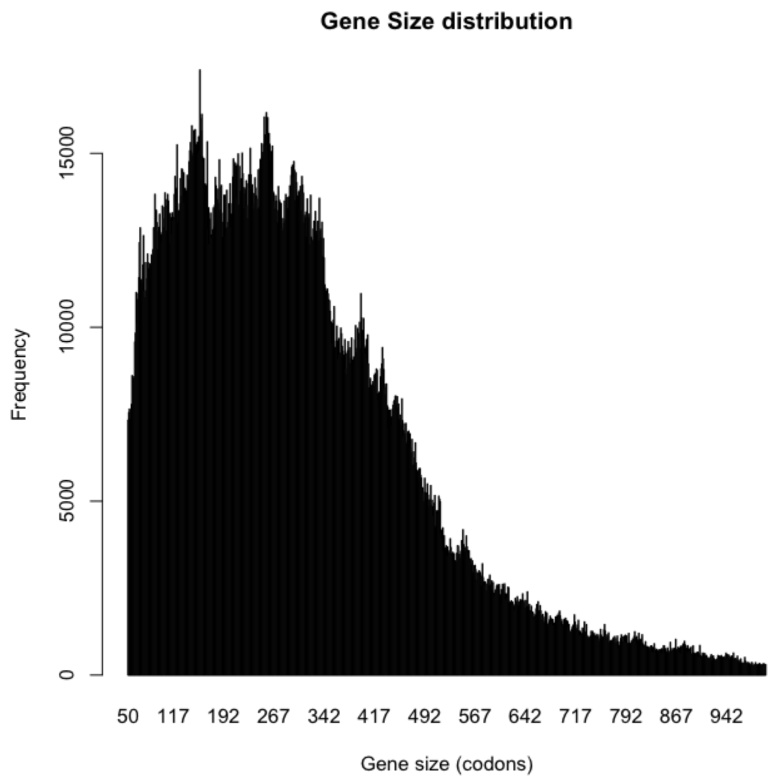

Figure S2 Prokaryotic Gene Size Empirical Distribution. In this figure, only gene sizes shorter than 1000 codons (3000 bp) are shown. The gene sizes used in the simulations were retrieved from this distribution.

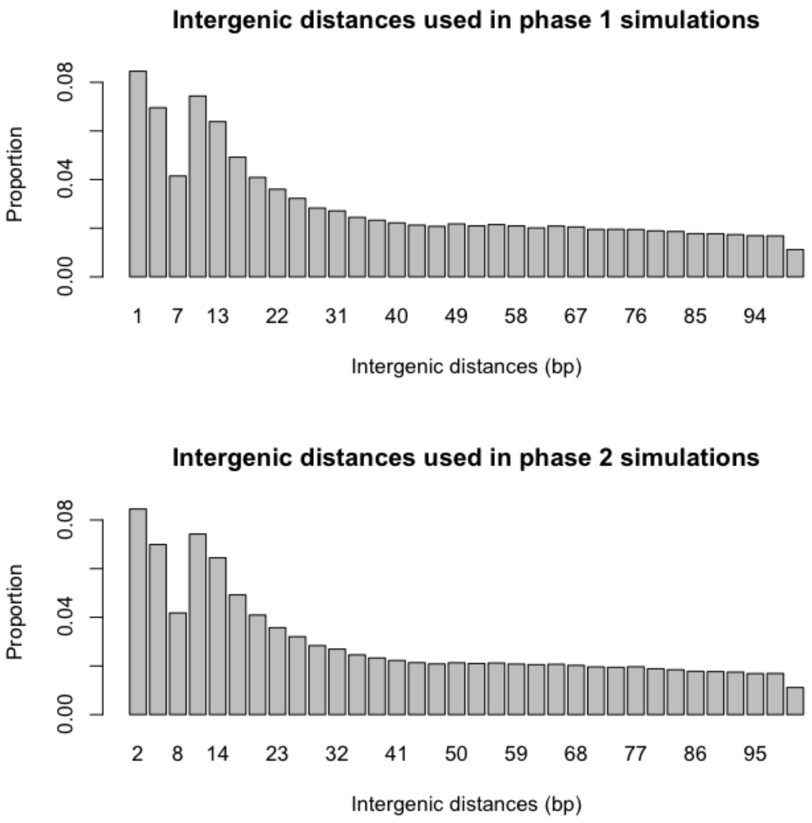

Figure S3 Intergenic Distances used in phase 1 and phase 2 simulations (scenarios 2 and 3). The values presented in these barplots were retrieved from the prokaryotic empirical intergenic distances distribution (figure S1). We have limited the distance up to 99 + phase bp for practical reasons.

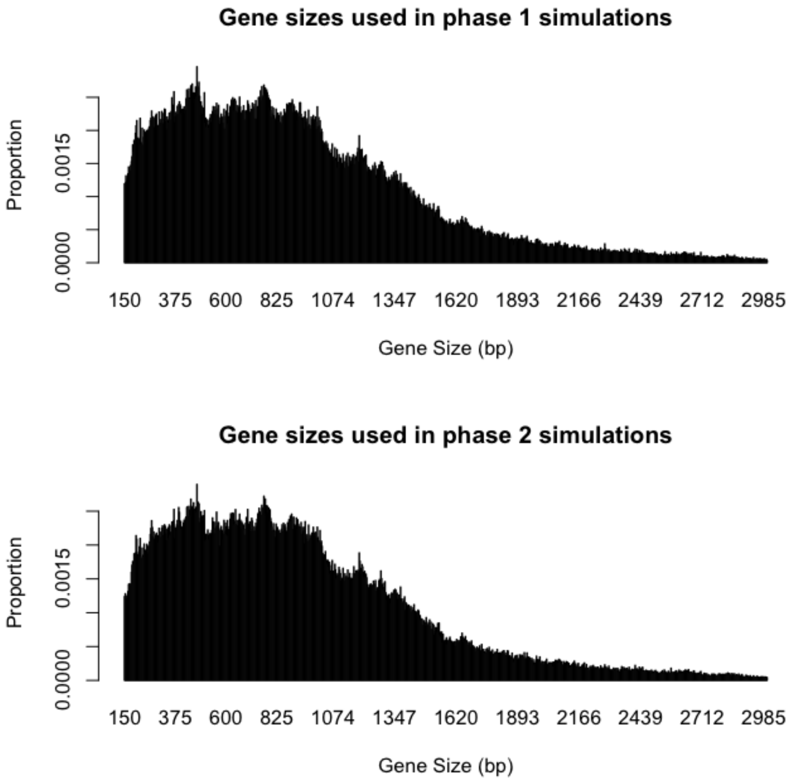

Figure S4 Gene sizes used in the simulations (scenarios 2 and 3). The values used are not dependent of the simulated phase.

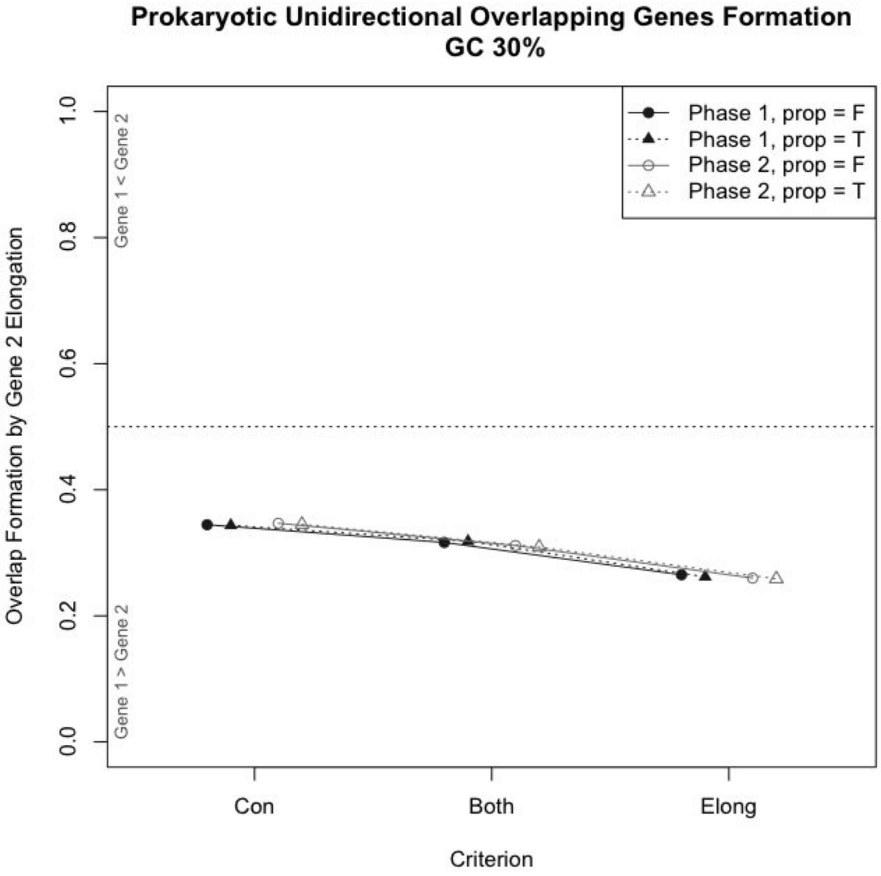

Figure S5 Proportion of overlaps caused by the elongation of gene 2. Data shown corresponds to the 30% GC content scenario. The simulations were run separately with three criteria: preference for gene 2 contraction (criterion "Con"); gene 2 elongation/contraction equally probable (criterion "Both"); preference for gene 2 elongation (criterion "Elong"). "Prop = F", start codons were chosen at random, "Prop = T", start codons chosen according to empirical codon usage in prokaryotic genomes (80% ATG, 17% GTG and 3% TTG). Each scenario was replicated 10^6 times. In all simulated scenarios, the formation of overlapping regions originated by the elongation of the 3'-end of gene were significantly more frequent than those originated by the elongation of gene 2 (*p*-values ≤ 0.001).

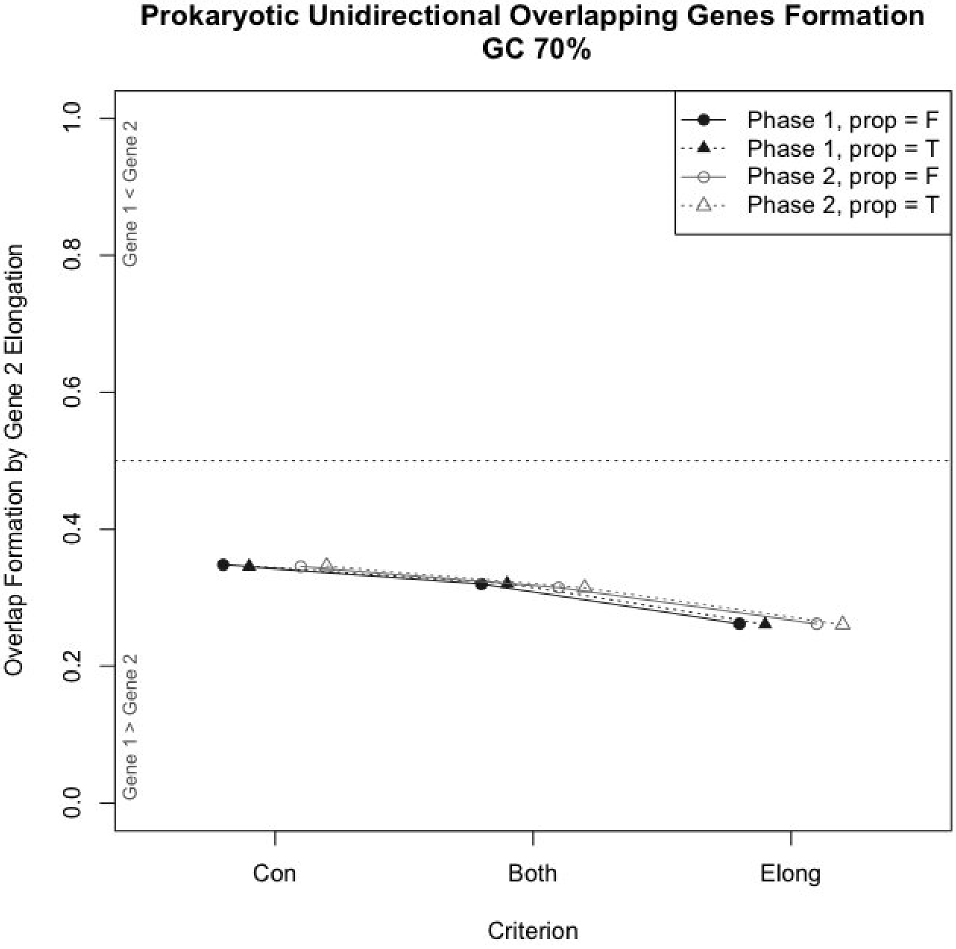

Figure S6 Proportion of overlaps caused by the elongation of gene 2. Data shown corresponds to the 70% GC content scenario. The simulations were run separately with three criteria: preference for gene 2 contraction (criterion “Con”); gene 2 elongation/contraction equally probable (criterion “Both”); preference for gene 2 elongation (criterion “Elong”). “Prop = F”, start codons were chosen at random, “Prop = T”, start codons chosen according to empirical codon usage in prokaryotic genomes (80% ATG, 17% GTG and 3% TTG). Each scenario was replicated 10^6 times. In all simulated scenarios, the formation of overlapping regions originated by the elongation of the 3’-end of gene were significantly more frequent than those originated by the elongation of gene 2 (*p*-values ≤ 0.001).

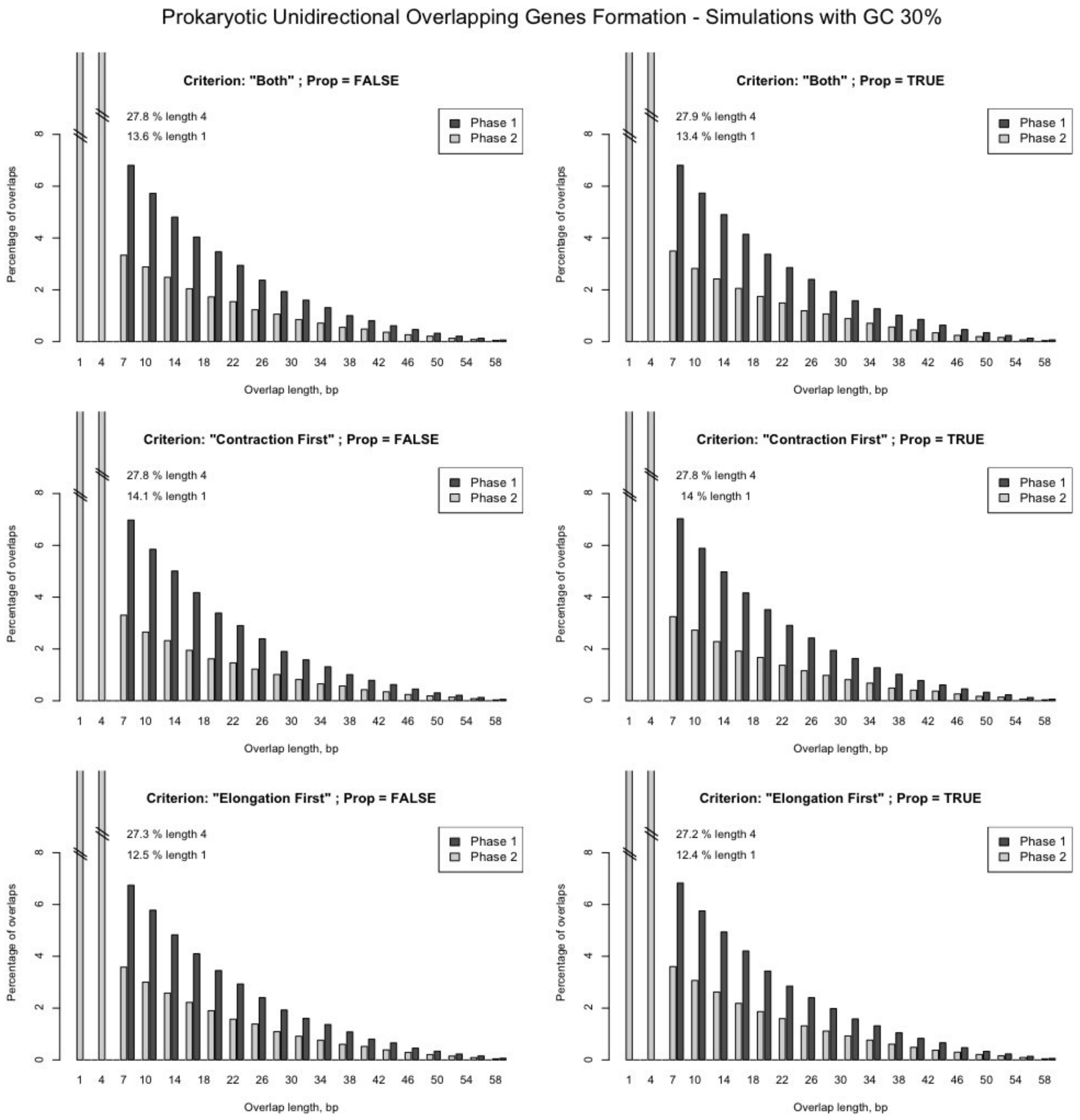

Figure S7 Hypothetical prokaryotic overlap lengths of unidirectional adjacent genes, calculated from simulated dataset (scenario 1). First set of simulations where gene size and intergenic distances were set to 63 bp and 60 bp + phase, respectively. Parameters: GC content = 30%; and all possible combinations between *criterion* ("Elongation First", "Both", "Contraction First") and *Proportions of start codons* (TRUE or FALSE). Frequency of overlaps in both phases was weighted according to the mutation rate of phase 1 and phase 2 simulations (see Material and Methods).

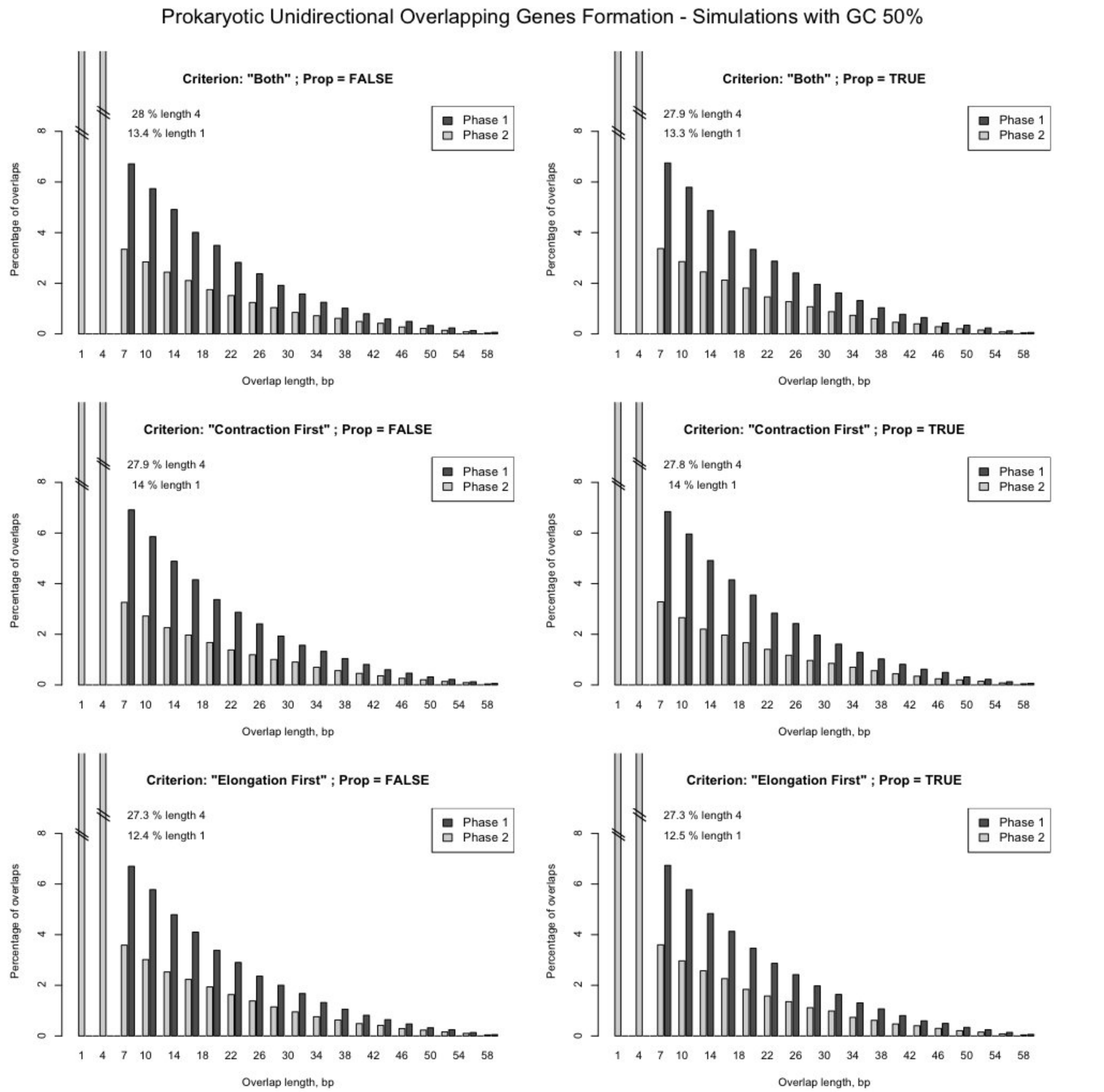

Figure S8 Hypothetical prokaryotic overlap lengths of unidirectional adjacent genes, calculated from simulated dataset (scenario 1). First set of simulations where gene size and intergenic distances were set to 63 bp and 60 bp + phase, respectively. Parameters: GC content = 50%; and all possible combinations between *criterion* ("Elongation First", "Both", "Contraction First") and *Proportions of start codons* (TRUE or FALSE). Frequency of overlaps in both phases was weighted according to the mutation rate between phase 1 and phase 2 simulations (see Material and Methods).

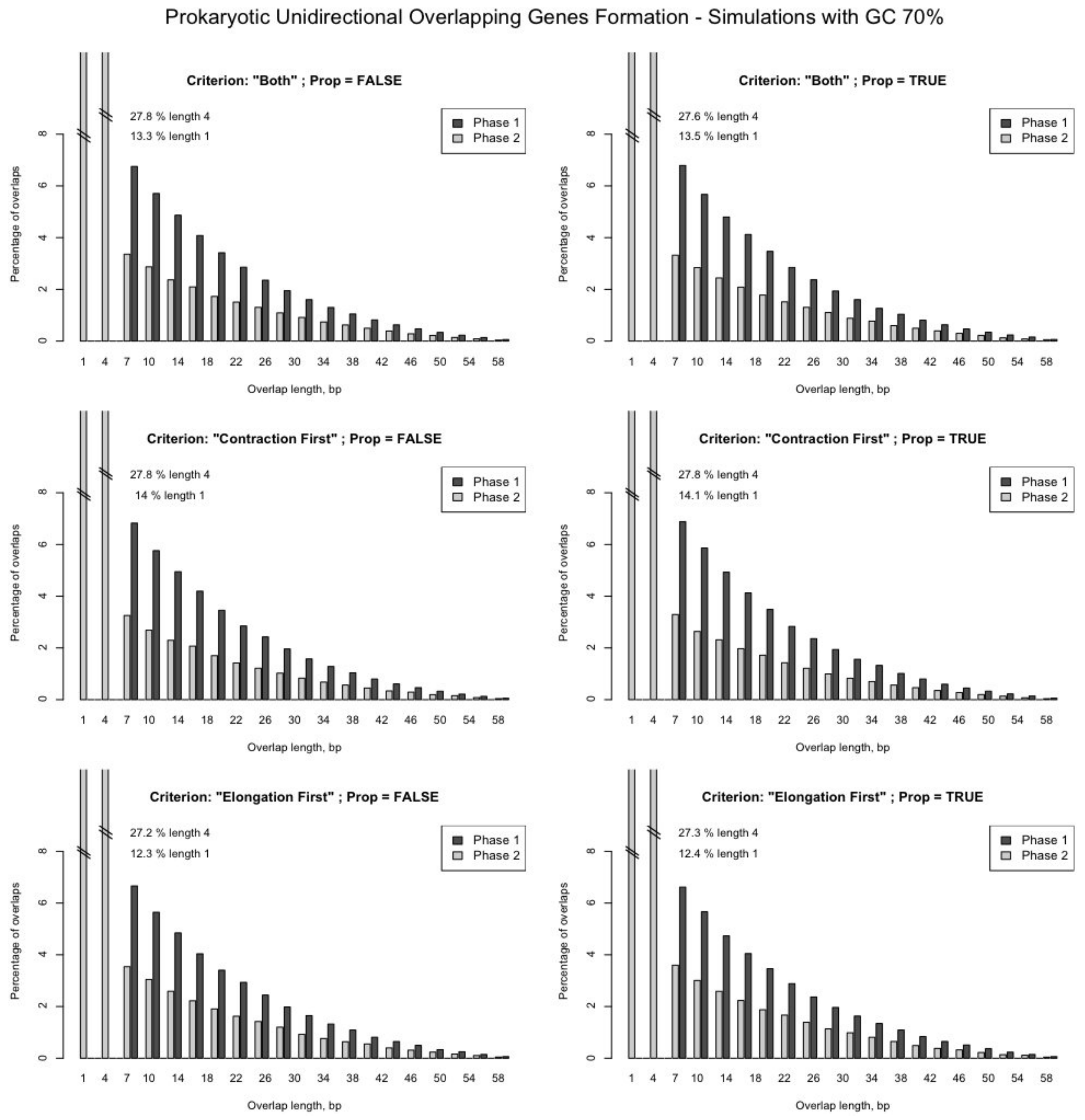

Figure S9 Hypothetical prokaryotic overlap lengths of unidirectional adjacent genes, calculated from simulated dataset (scenario 1). First set of simulations where gene size and intergenic distances were set to 63 bp and 60 bp + phase, respectively. Parameters: GC content = 70%; and all possible combinations between *criterion* ("Elongation First", "Both", "Contraction First") and *Proportions of start codons* (TRUE or FALSE). Frequency of overlaps in both phases was weighted according to the mutation rate between phase 1 and phase 2 simulations (see Material and Methods).

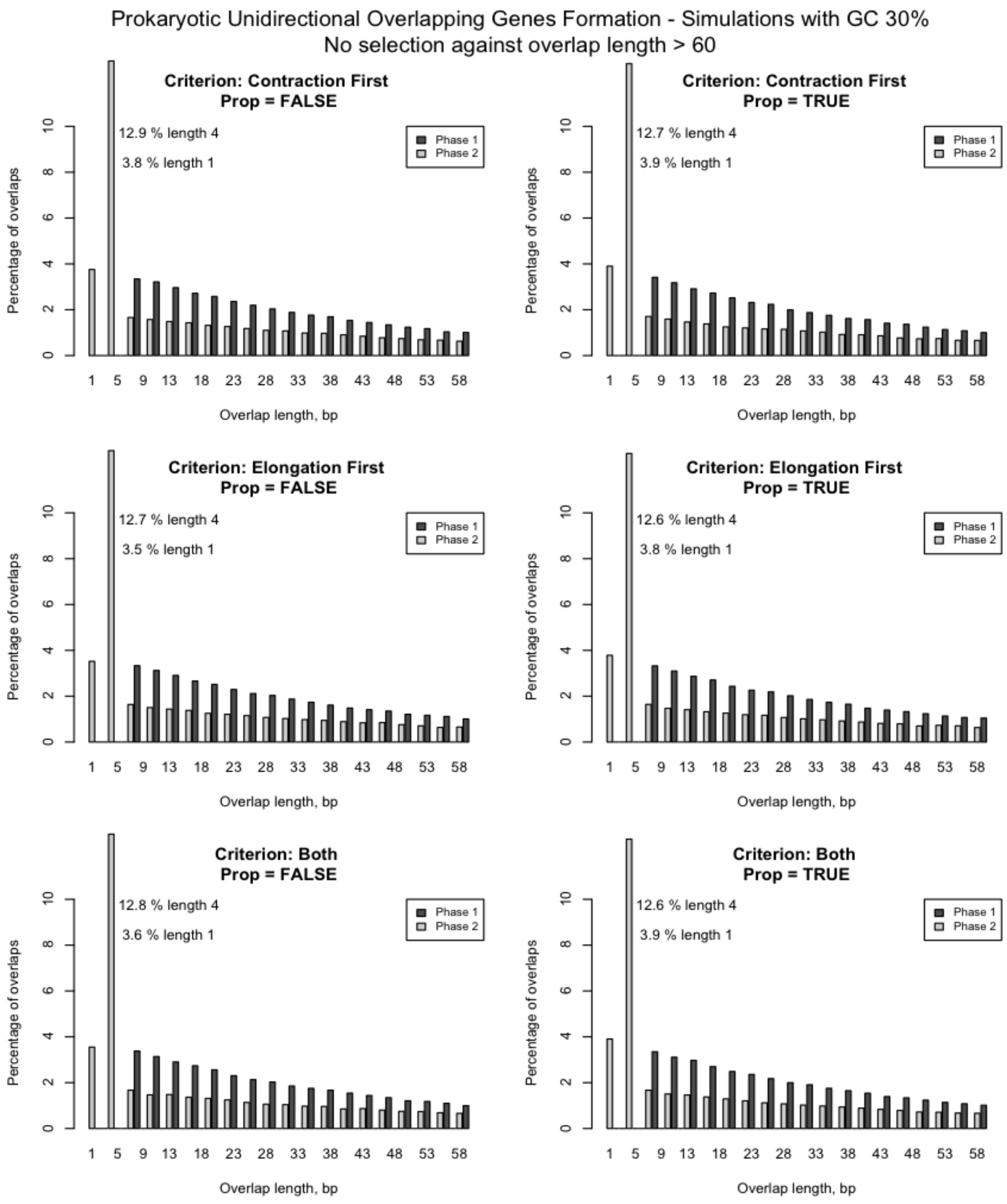

Figure S10 Hypothetical prokaryotic overlap lengths of unidirectional adjacent genes, calculated from simulated dataset (scenario 2). Second set of simulations where gene size and intergenic distances were retrieved from an empirical distribution of prokaryotic genomes (see Figures S1-S4). Parameters: GC content = 30%; and all possible combinations between *criterion* ("Elongation First", "Both", "Contraction First") and *Proportions of start codons* (TRUE or FALSE). No weighting scheme was applied to the representativeness of phase 1 or phase 2. No selection against overlap length > 60 bp was included. Barplot is limited to show only overlap length < 60 bp.

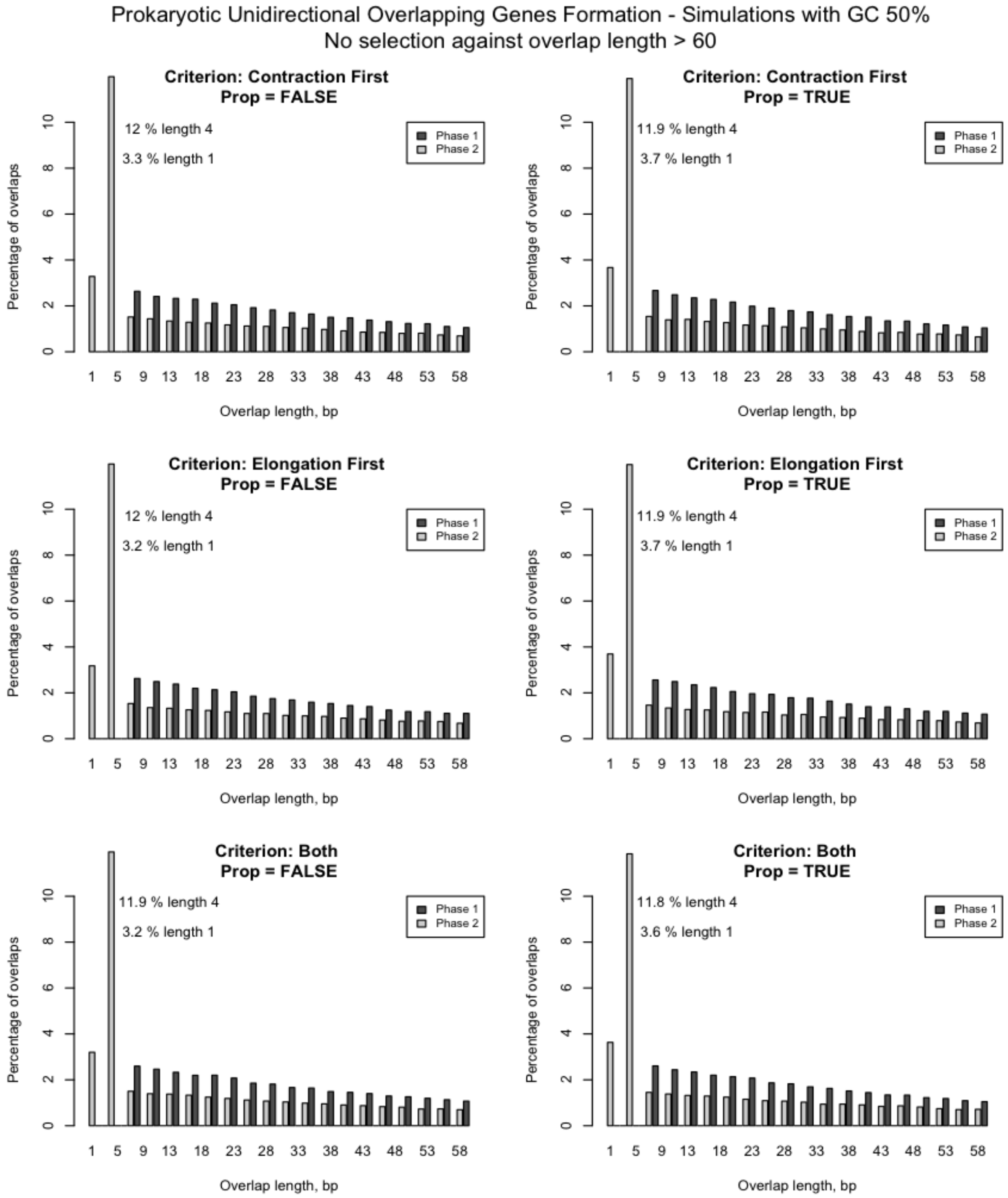

Figure S11 Hypothetical prokaryotic overlap lengths of unidirectional adjacent genes, calculated from simulated dataset (scenario 2). Second set of simulations where gene size and intergenic distances were retrieved from an empirical distribution of prokaryotic genomes (see Figures S1-S4). Parameters: GC content = 50%; and all possible combinations between *criterion* ("Elongation First", "Both", "Contraction First") and *Proportions of start codons* (TRUE or FALSE). No weighting scheme was applied to the representativeness of phase 1 or phase 2. No selection against overlap length > 60 bp was included. Barplot is limited to show only overlap length < 60 bp.

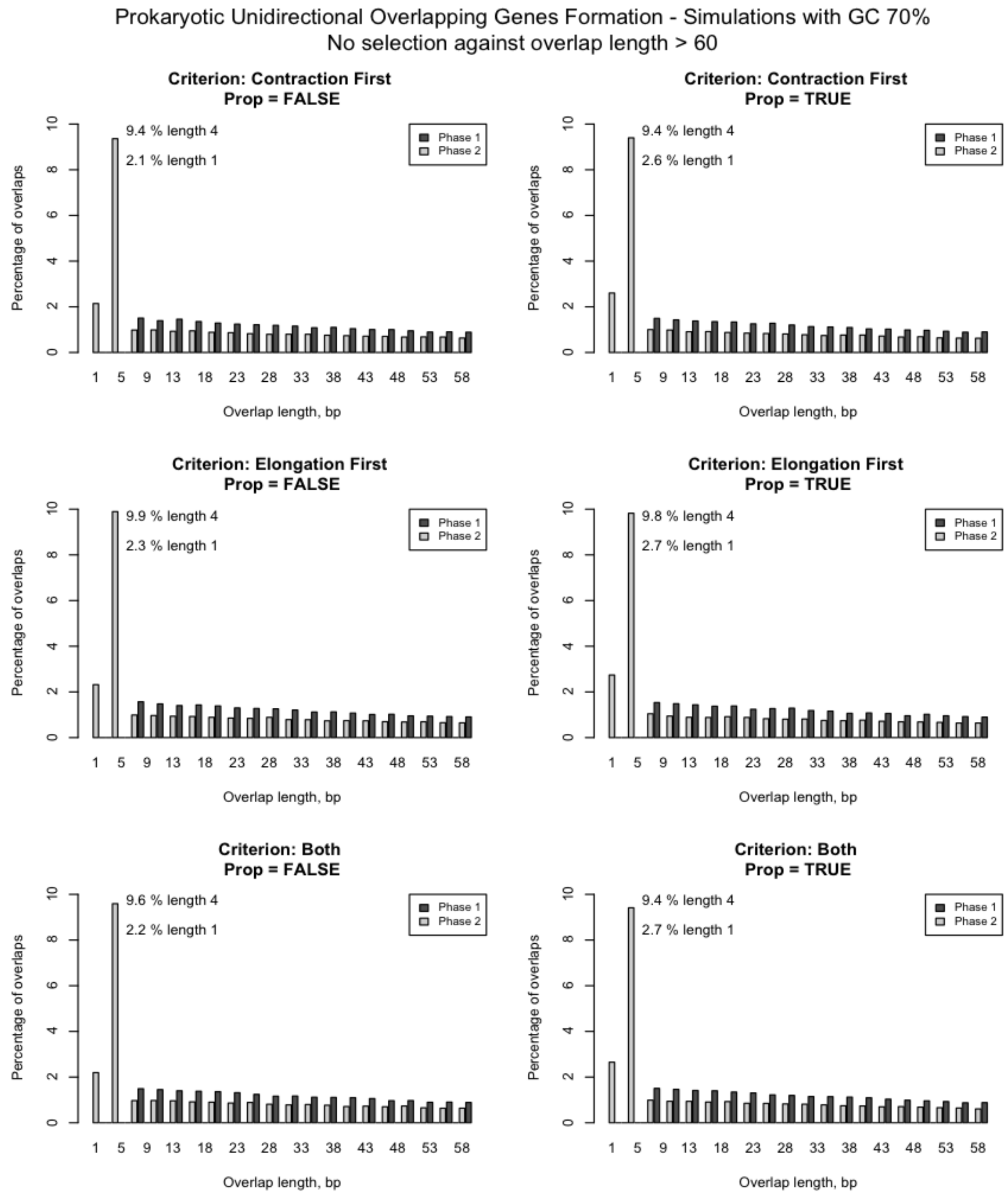

Figure S12 Hypothetical prokaryotic overlap lengths of unidirectional adjacent genes, calculated from simulated dataset (scenario 2). Second set of simulations where gene size and intergenic distances were retrieved from an empirical distribution of prokaryotic genomes (see Figures S1-S4). Parameters: GC content = 70%; and all possible combinations between *criterion* ("Elongation First", "Both", "Contraction First") and *Proportions of start codons* (TRUE or FALSE). No weighting scheme was applied to the representativeness of phase 1 or phase 2. No selection against overlap length > 60 bp was included. Barplot is limited to show only overlap length < 60 bp.

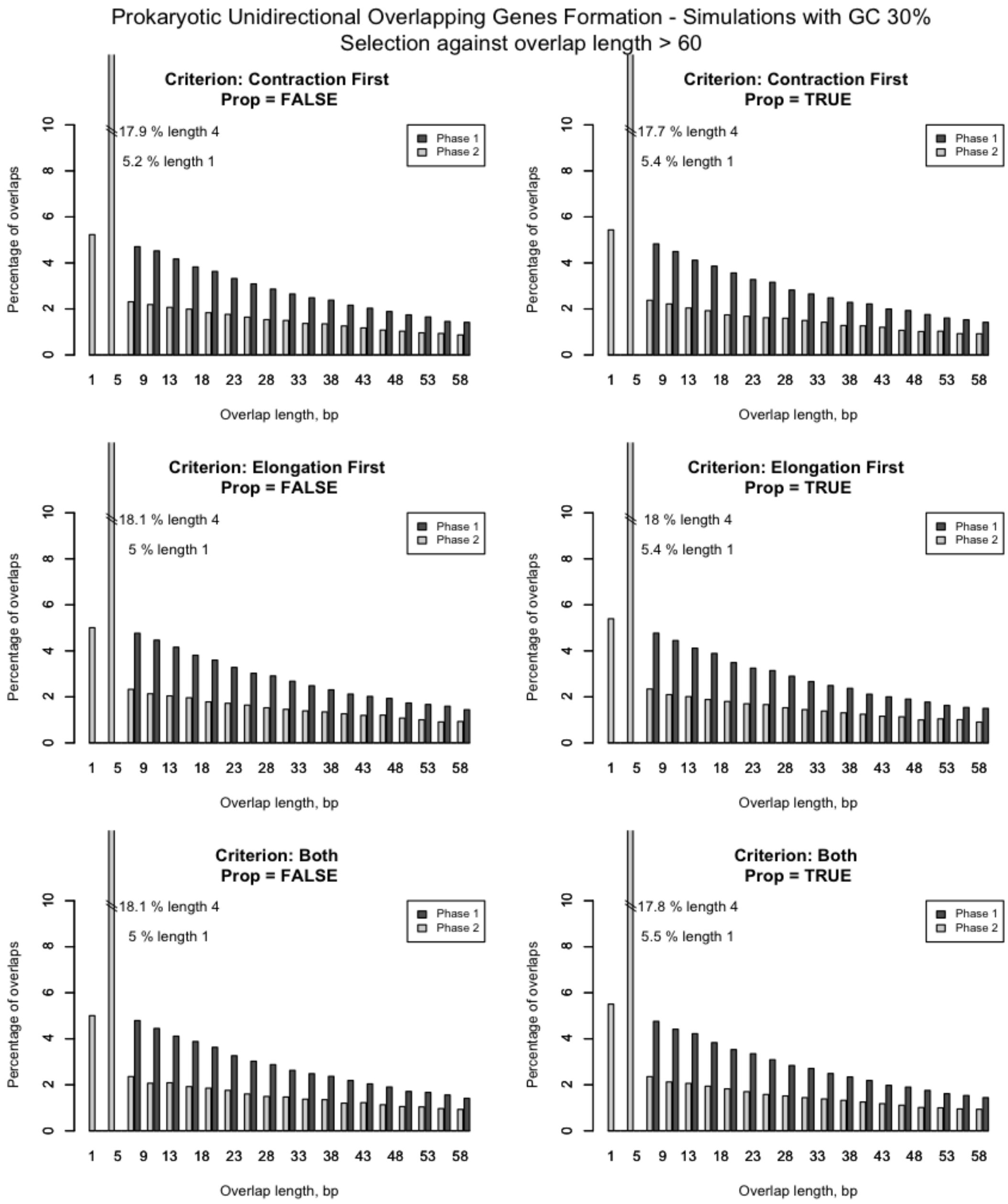

Figure S13 Hypothetical prokaryotic overlap lengths of unidirectional adjacent genes, calculated from simulated dataset (scenario 3). Third set of simulations where gene size and intergenic distances were retrieved from an empirical distribution of prokaryotic genomes (see Figures S1-S4). Parameters: GC content = 30%; and all possible combinations between *criterion* ("Elongation First", "Both", "Contraction First") and *Proportions of start codons* (TRUE or FALSE). No weighting scheme was applied to the representativeness of phase 1 or phase 2. Selection against overlap length > 60 bp was included.

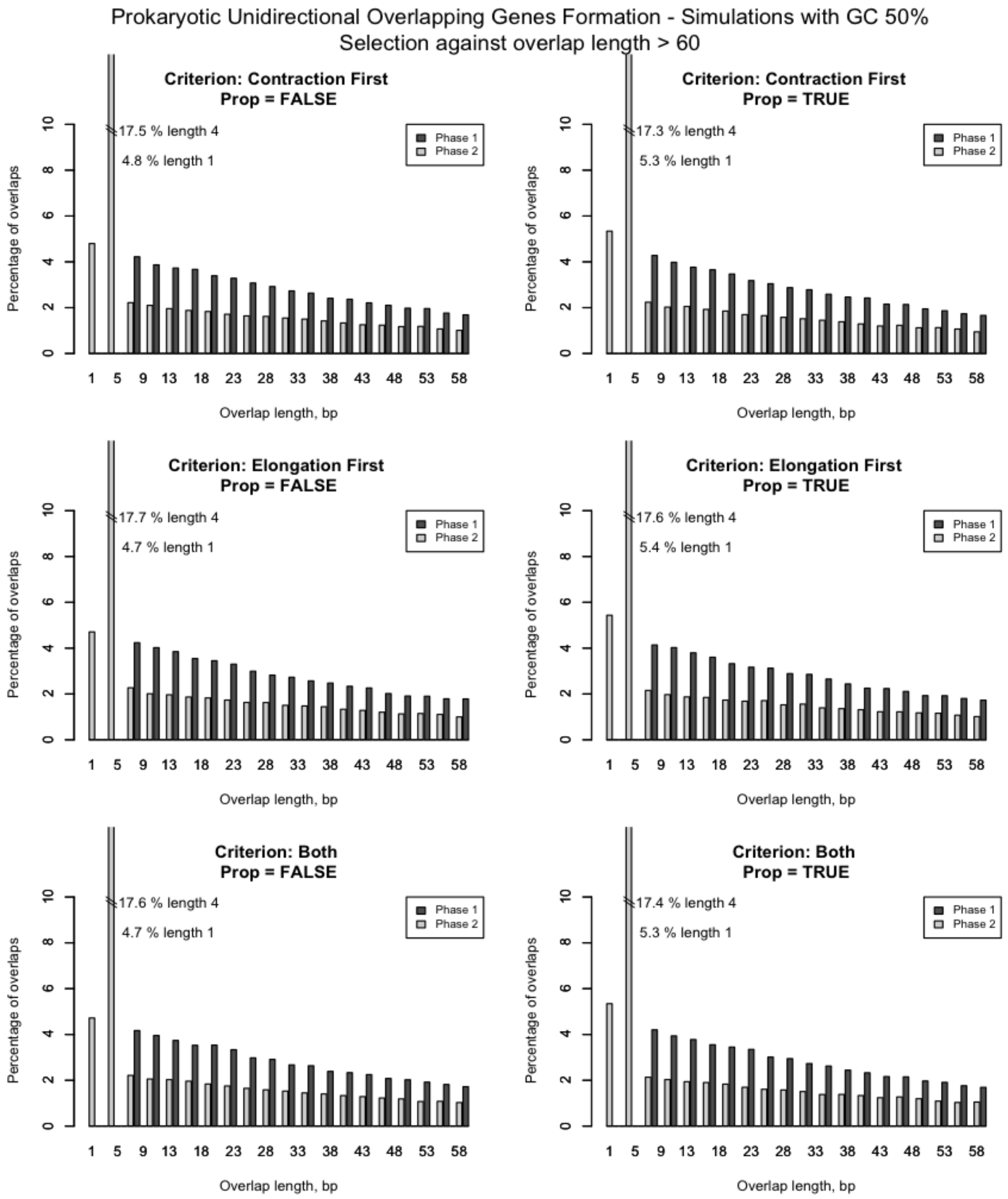

Figure S14 Hypothetical prokaryotic overlap lengths of unidirectional adjacent genes, calculated from simulated dataset (scenario 3). Third set of simulations where gene size and intergenic distances were retrieved from an empirical distribution of prokaryotic genomes (see Figures S1-S4). Parameters: GC content = 50%; and all possible combinations between *criterion* (“Elongation First”, “Both”, “Contraction First”) and *Proportions of start codons* (TRUE or FALSE). No weighting scheme was applied to the representativeness of phase 1 or phase 2. Selection against overlap length > 60 bp was included.

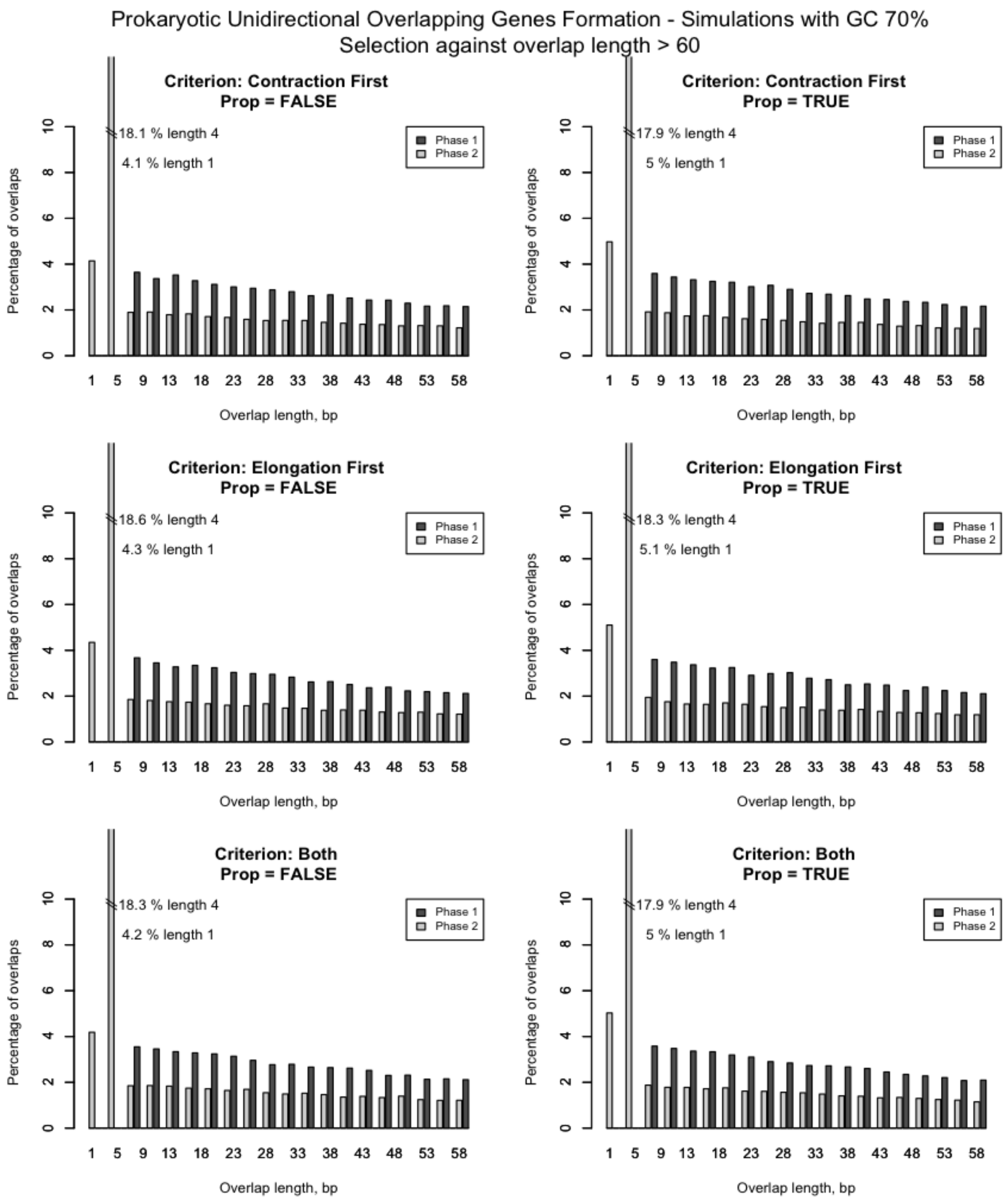

Figure S15 Hypothetical prokaryotic overlap lengths of unidirectional adjacent genes, calculated from simulated dataset (scenario 3). Third set of simulations where gene size and intergenic distances were retrieved from an empirical distribution of prokaryotic genomes (see Figures S1-S4). Parameters: GC content = 70%; and all possible combinations between *criterion* ("Elongation First", "Both", "Contraction First") and *Proportions of start codons* (TRUE or FALSE). No weighting scheme was applied to the representativeness of phase 1 or phase 2. Selection against overlap length > 60 bp was included.

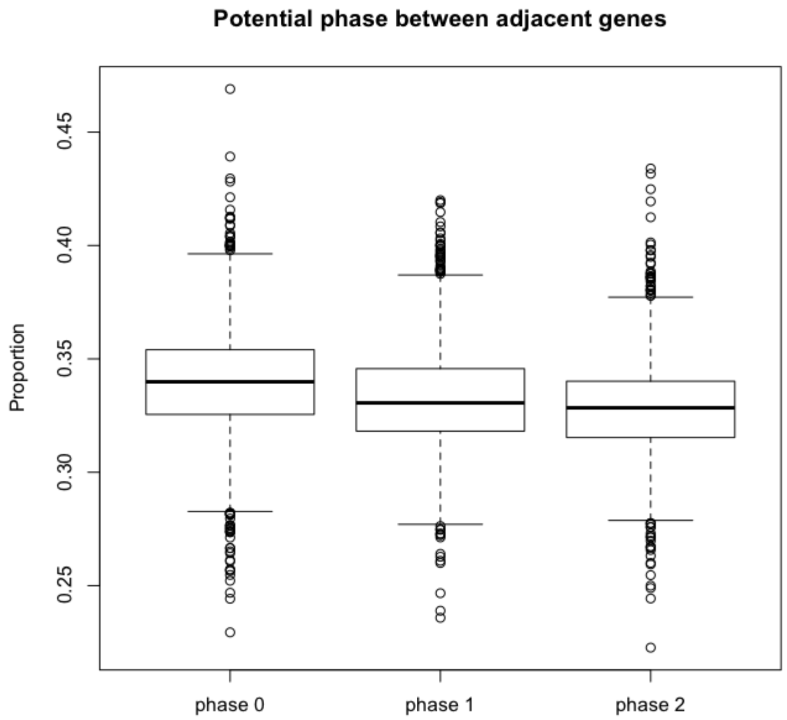

Figure S16 Potential overlapping phase between adjacent non-overlapping gene pairs. We measured the potential overlapping phase between neighboring non-overlapping genes separated by 200 bp or less. The proportions of each phase is near 1/3, although phase 0 > phase 1 > phase2.

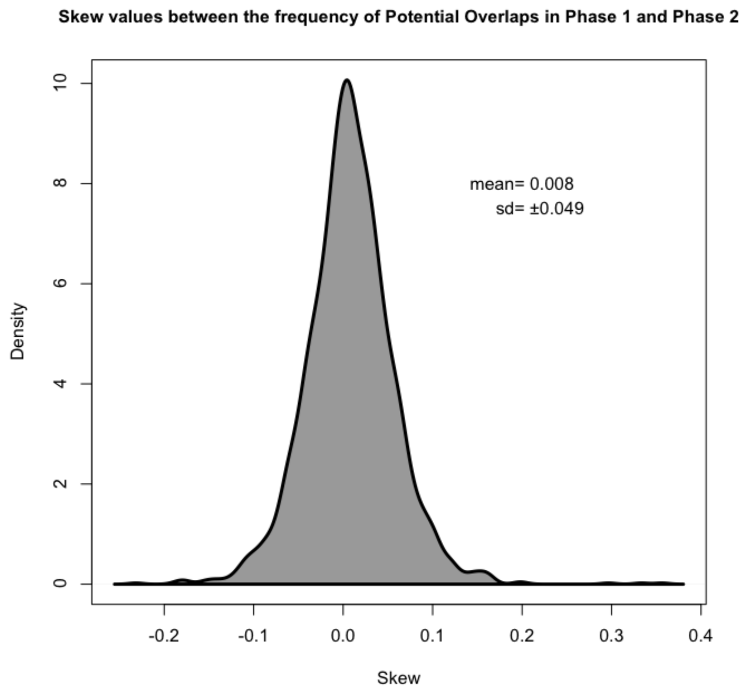

Figure S17 Distribution of the skew values for the pairwise differences between potential phase 1 and potential phase 2 overlaps. Skew values were calculated for each genome as followed: *(fpp1 – fpp2)/(fpp1 + fpp2)*, where *fpp1*: frequency of potential phase 1 overlaps and *fpp2*: frequency of potential phase 1 overlaps. Skew values can vary between -1 (no potential phase 2 overlaps) and 1 (no potential phase 1 overlaps). If skew equals to zero, then no biased distribution is found. Our results show a small mean skewed distribution towards potential phase 1 overlaps (mean = 0.008, sd = ±0.049).

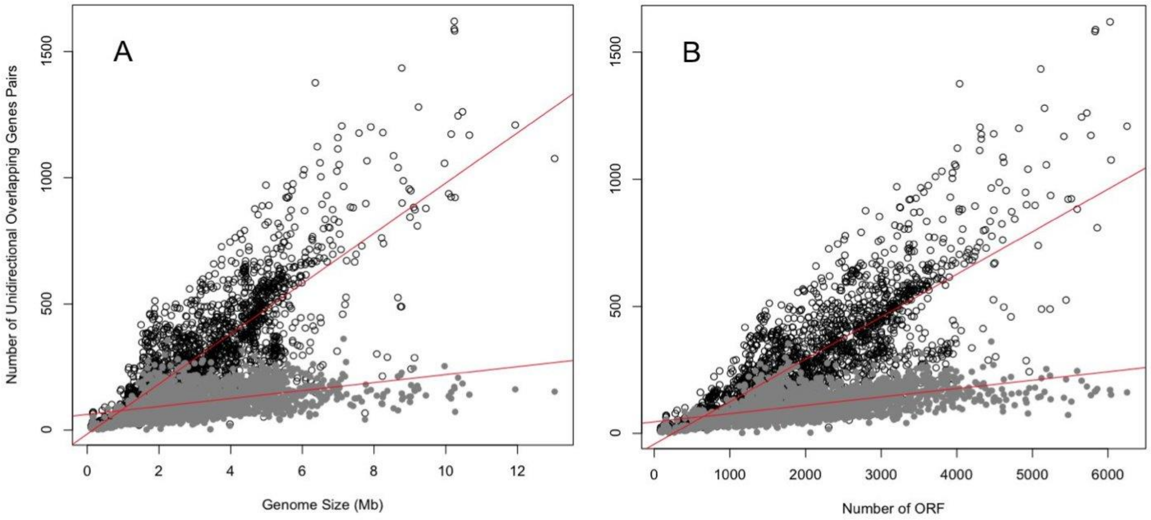

Figure S18 (A) Relationship between genome length and number of unidirectional overlapping genes pairs. Each point represents an individual genome. Closed and open circles correspond to overlapping genes pairs in phase 1 and phase 2, respectively. Linear regression (phase 1), $r^2 = 0.23$, p-value < 0.001. Linear regression (phase 2), $r^2 = 0.64$, p-value < 0.001.
(B) Relationship between total number of unidirectional pairs of open reading frames (ORF) and the number of unidirectional overlapping genes pairs. Each point represents an individual genome. Closed and open circles correspond to overlapping genes pairs in phase 1 and phase 2, respectively. Linear regression (phase 1), $r^2 = 0.35$, p-value < 0.001. Linear regression (phase 2), $r^2 = 0.63$, p-value < 0.001.

It was shown previously that the total number of ORF increases linearly with prokaryotic genome size (Mira *et al.* 2001, Fukuda *et al.* 2003). The same relationship was reported for the number of overlapping genes pairs and the genome size (Fukuda *et al.* 2003). Here, using 2,151 prokaryotic genomes, we show that these correlations are also recovered, when unidirectional overlapping genes pairs are separated into phase 1 and 2 (Figure S18 A). Additionally, we show a linear correlation of the total number of unidirectional neighboring genes pairs (potential but not overlapping genes, intergenic distance up to 100 nucleotides) and the total number of unidirectional overlapping genes (Figure S18 B). These results corroborate the hypothesis that both phase 1 and phase 2 overlapping genes have a uniform formation rate across species (Fukuda *et al.* 2003). However, it should be highlighted that prokaryotic genomes are not independent from each other, as they share an evolutionary history, and hence, these results should be analyzed with caution.

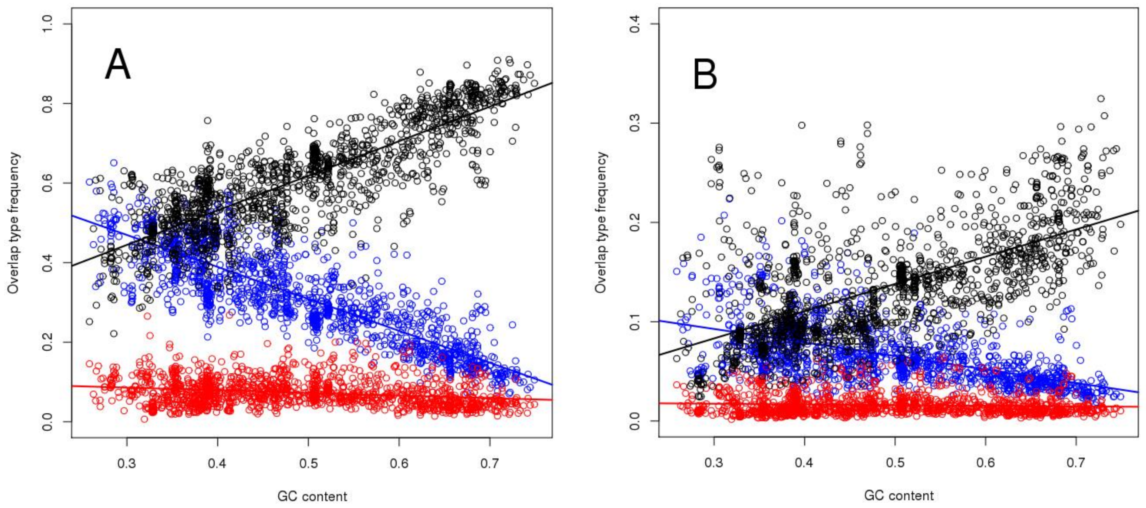

Figure S19 Relative frequency of overlapping genes in 1453 prokaryotic genomes plotted against genomic GC content. Short phase 2 overlaps are plotted in black, long phase 1 overlaps in blue and long phase-2 overlaps are plotted in red. The frequency of short phase 2 overlaps is positively correlated with genomic GC content ((A), $r^2 = 0.69$, $p < 0.001$; (B), $r^2 = 0.38$, $p < 0.001$). In contrast, the correlation between the frequency of long phase 1 and phase 2 overlaps and GC content is significantly negative (long phase 1: (A), $r^2 = 0.72$, $p < 0.001$ and (B), $r^2 = 0.31$, $p < 0.001$; long phase 2: (A) $r^2 = 0.06$, $p < 0.001$). Overlap percentages refer to the proportion of each overlap type (short phase 2, long phase 1 or long phase 2) (A) among all real overlapping genes or (B) among all real and potential overlapping genes pairs. Only genomes with 100 or more overlapping genes were considered in this analysis.

REFERENCES

Fukuda Y., Nakayama Y., Tomita M., 2003 On dynamics of overlapping genes in bacterial genomes. *Gene* **323**: 181–187.

Mira A., Ochman H., Moran M. A., 2001 Deletional bias and the evolution of bacterial genomes. *Trends Genet.* **17**: 589–596.

Table S1 Proportions of prokaryotic unidirectional overlapping genes in phase 1 and phase 2. Overall proportions of phase 1 and phase 2 overlapping genes pairs (OGP) are shown on the left side of the table and proportions of long phase 1 and phase 2 OGP on the right side. All taxon specific OGP were filtered from the non-redundant OGP database. Only unique overlap lengths for each homologous OGP were used in the comparison between long phase 1 and phase 2 overlaps (superscript a).

Taxonomic group	Phase 1 (%)	Phase 2 (%)	Number of overlapping genes pairs	Phase 1 long (%)	Phase 2 long (%)	Number of overlapping genes pairs[a]
Archaea						14,243
Crenarchaeota	37.5***	62.5	8,863	73.2***	26.8	5,749
Euryarchaeota	31.7***	68.3	16,696	77.6***	22.4	8,190
Korarchaeota	37.1***	62.9	321	88.3***	11.7	137
Nanoarchaeota	35.7***	64.3	143	71.8***	28.2	71
Thaumarchaeota	19.2***	80.8	442	88.5***	11.5	96
Bacteria						155,633
Acidobacteria	21.1***	78.9	4,315	78.9***	21.1	1,192
Actinobacteria	16.6***	83.4	61,133	71.9***	28.1	15,989
Aquificae	36.4***	63.6	4,141	78.5***	21.5	2,081
Bacteroidetes	36.3***	63.7	17,541	81.9***	18.1	8,743
Chlamydiae	37.1***	62.9	1,950	70.7***	29.3	1,229
Chlorobi	27.8***	72.2	2,097	76.7***	23.3	853
Chloroflexi	27.4***	72.6	4,862	81.4***	18.6	1,773
Chrysiogenetes	26.3***	73.7	590	81.6***	18.4	190
Cyanobacteria	32.1***	67.9	7,039	75.3***	24.7	3,294
Deferribacteres	45.6***	54.4	2,413	86.4***	13.6	1,427
Deinococcus-Thermus	22.5***	77.5	5,574	73.5***	26.5	1,927
Dictyoglomi	39.3***	60.7	708	79.8***	20.2	372
Elusimicrobia	32.7***	67.3	376	79.9***	20.1	154
Fibrobacteres	29.1***	70.9	461	81.0***	19.0	174
Firmicutes	43.6***	56.4	50,413	83.0***	17.0	35,673
Fusobacteria	44.4***	55.6	1,411	86.4***	13.6	778
Gemmatimonadetes	14.7***	85.3	774	84.4***	15.6	135
Nitrospirae	27.1***	72.9	1,825	75.3***	24.7	665
Planctomycetes	21.7***	78.3	3,071	64.4***	35.6	1,047
Proteobacteria	27.6***	72.4	154,188	76.3***	23.7	68,774
Spirochaetes	33.5***	66.5	9,725	82.0***	18.0	4,469
Synergistetes	37.6***	62.4	1,520	78.3***	21.7	812
Tenericutes	45.6	54.4	507	91.1***	8.9	257

Thermobaculum	40.8***	59.2	463	84.9***	15.1	225
Thermotogae	35.7***	64.3	4,908	78.8***	21.2	2,870
Verrucomicrobia	27.7***	72.3	1,439	75.3***	24.7	530

****p* value ≤ 0.001
