# Supplementary material for: Origin and Length Distribution of Unidirectional Prokaryotic Overlapping Genes
Source: G3 (Bethesda). 2013 Nov 5;4(1):19–27. doi: 10.1534/g3.113.005652 (PMC3887535; doi:10.1534/g3.113.005652)
Supplement: Supporting Information [file supp_g3.113.005652_FigureS1.pdf]

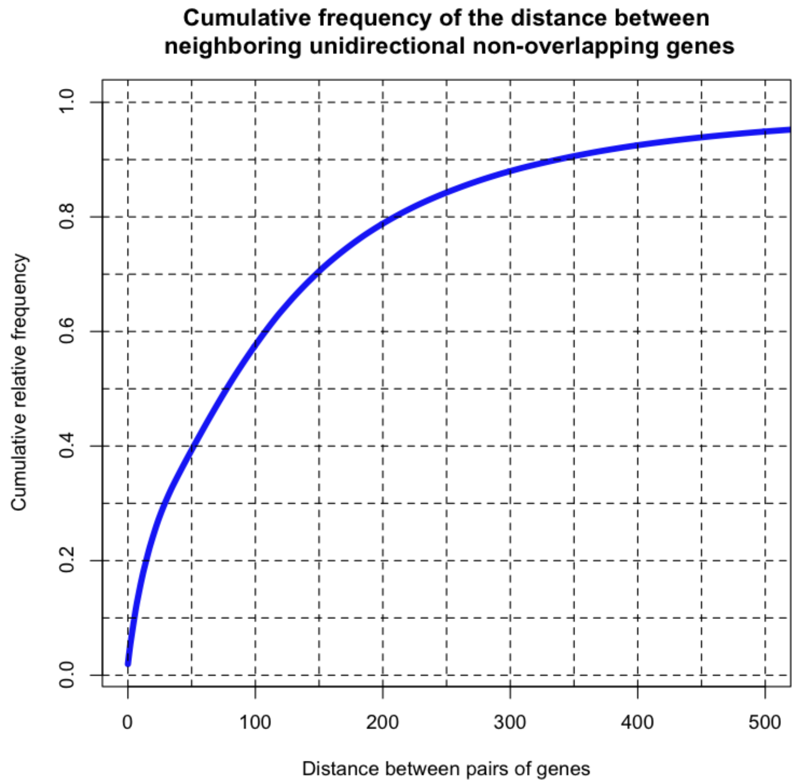

Figure S1 Cumulative frequency of the distance (in bp) between neighboring unidirectional non-overlapping genes. The figure shows intergenic distances up to 500 bp. The intergenic distances used in the simulations were retrieved from this empirical distribution. We have limited the distance up to 100 bp + phase for practical reasons. This interval [0-99 + phase] bp includes almost 60% of all empirical intergenic distances found between adjacent unidirectional genes in prokaryotic genomes.
