# Supplementary material for: Origin and Length Distribution of Unidirectional Prokaryotic Overlapping Genes
Source: G3 (Bethesda). 2013 Nov 5;4(1):19–27. doi: 10.1534/g3.113.005652 (PMC3887535; doi:10.1534/g3.113.005652)
Supplement: Supporting Information [file supp_g3.113.005652_FigureS2.pdf]

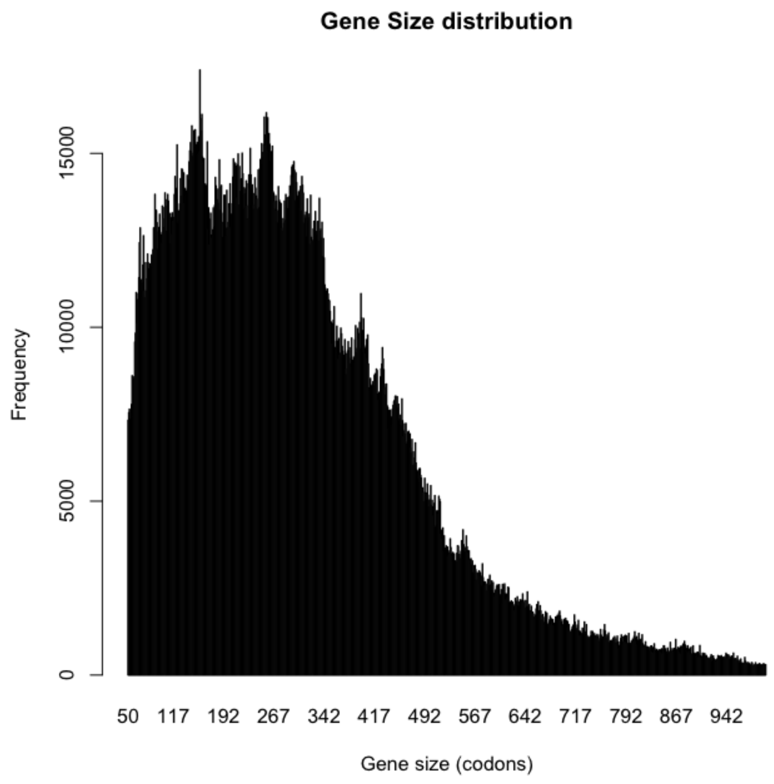

Figure S2 Prokaryotic Gene Size Empirical Distribution. In this figure, only gene sizes shorter than 1000 codons (3000 bp) are shown. The gene sizes used in the simulations were retrieved from this distribution.
