# Supplementary material for: Origin and Length Distribution of Unidirectional Prokaryotic Overlapping Genes
Source: G3 (Bethesda). 2013 Nov 5;4(1):19–27. doi: 10.1534/g3.113.005652 (PMC3887535; doi:10.1534/g3.113.005652)
Supplement: Supporting Information [file supp_g3.113.005652_FigureS3.pdf]

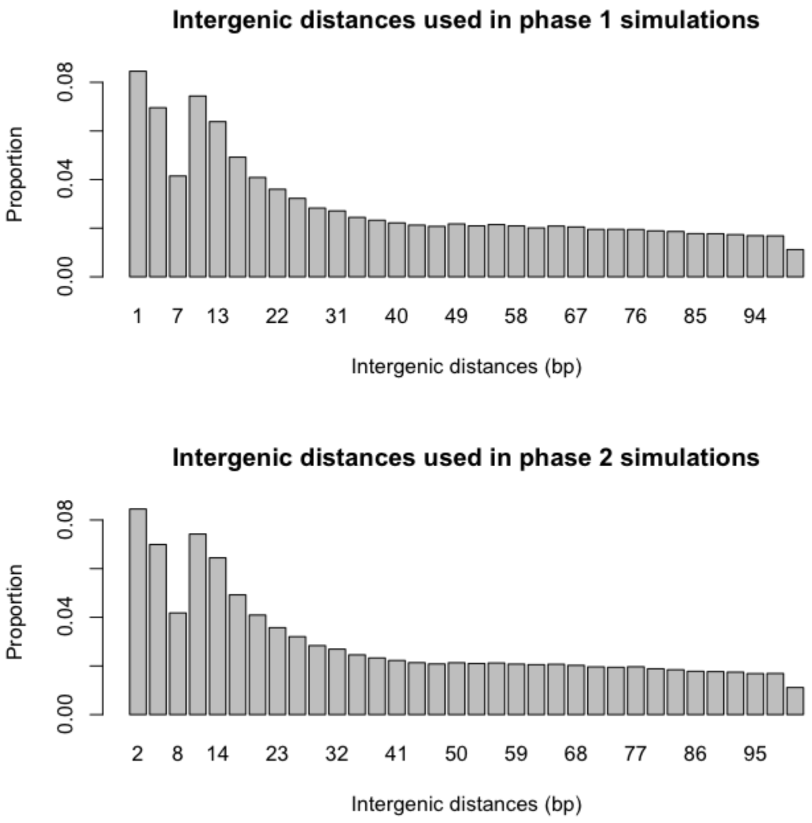

Figure S3 Intergenic Distances used in phase 1 and phase 2 simulations (scenarios 2 and 3). The values presented in these barplots were retrieved from the prokaryotic empirical intergenic distances distribution (figure S1). We have limited the distance up to 99 + phase bp for practical reasons.
