# Supplementary material for: Origin and Length Distribution of Unidirectional Prokaryotic Overlapping Genes
Source: G3 (Bethesda). 2013 Nov 5;4(1):19–27. doi: 10.1534/g3.113.005652 (PMC3887535; doi:10.1534/g3.113.005652)
Supplement: Supporting Information [file supp_g3.113.005652_FigureS5.pdf]

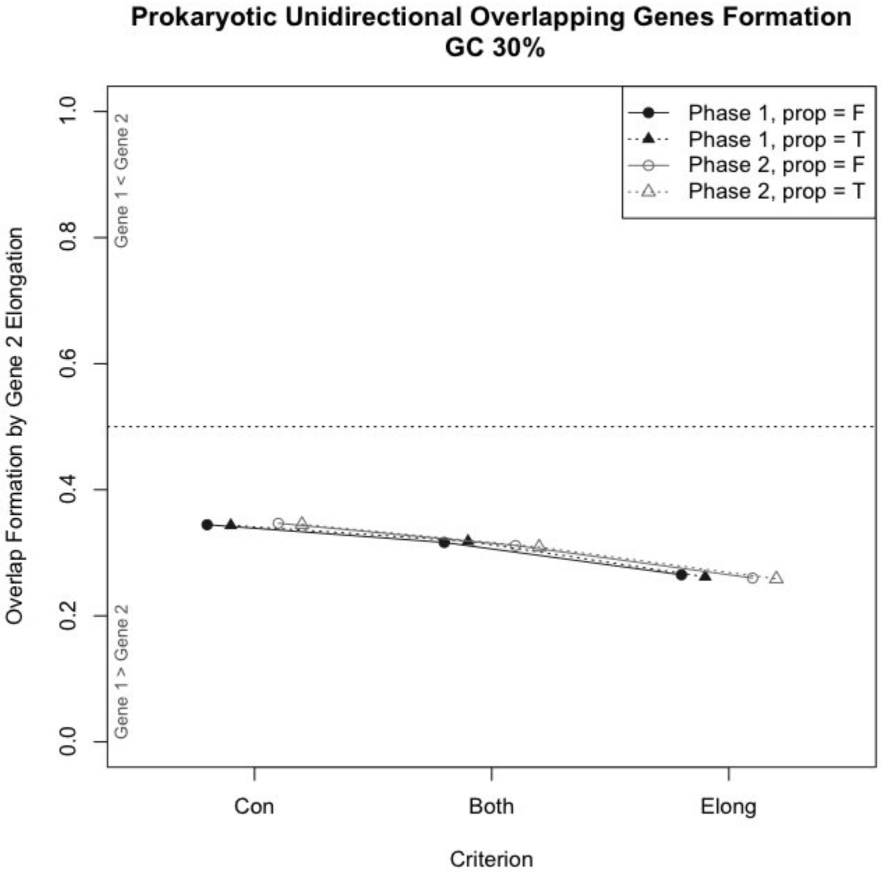

Figure S5 Proportion of overlaps caused by the elongation of gene 2. Data shown corresponds to the 30% GC content scenario. The simulations were run separately with three criteria: preference for gene 2 contraction (criterion "Con"); gene 2 elongation/contraction equally probable (criterion "Both"); preference for gene 2 elongation (criterion "Elong"). "Prop = F", start codons were chosen at random, "Prop = T", start codons chosen according to empirical codon usage in prokaryotic genomes (80% ATG, 17% GTG and 3% TTG). Each scenario was replicated 10^6 times. In all simulated scenarios, the formation of overlapping regions originated by the elongation of the 3'-end of gene were significantly more frequent than those originated by the elongation of gene 2 (p-values ≤ 0.001).
