# Supplementary material for: Origin and Length Distribution of Unidirectional Prokaryotic Overlapping Genes
Source: G3 (Bethesda). 2013 Nov 5;4(1):19–27. doi: 10.1534/g3.113.005652 (PMC3887535; doi:10.1534/g3.113.005652)
Supplement: Supporting Information [file supp_g3.113.005652_FigureS7.pdf]

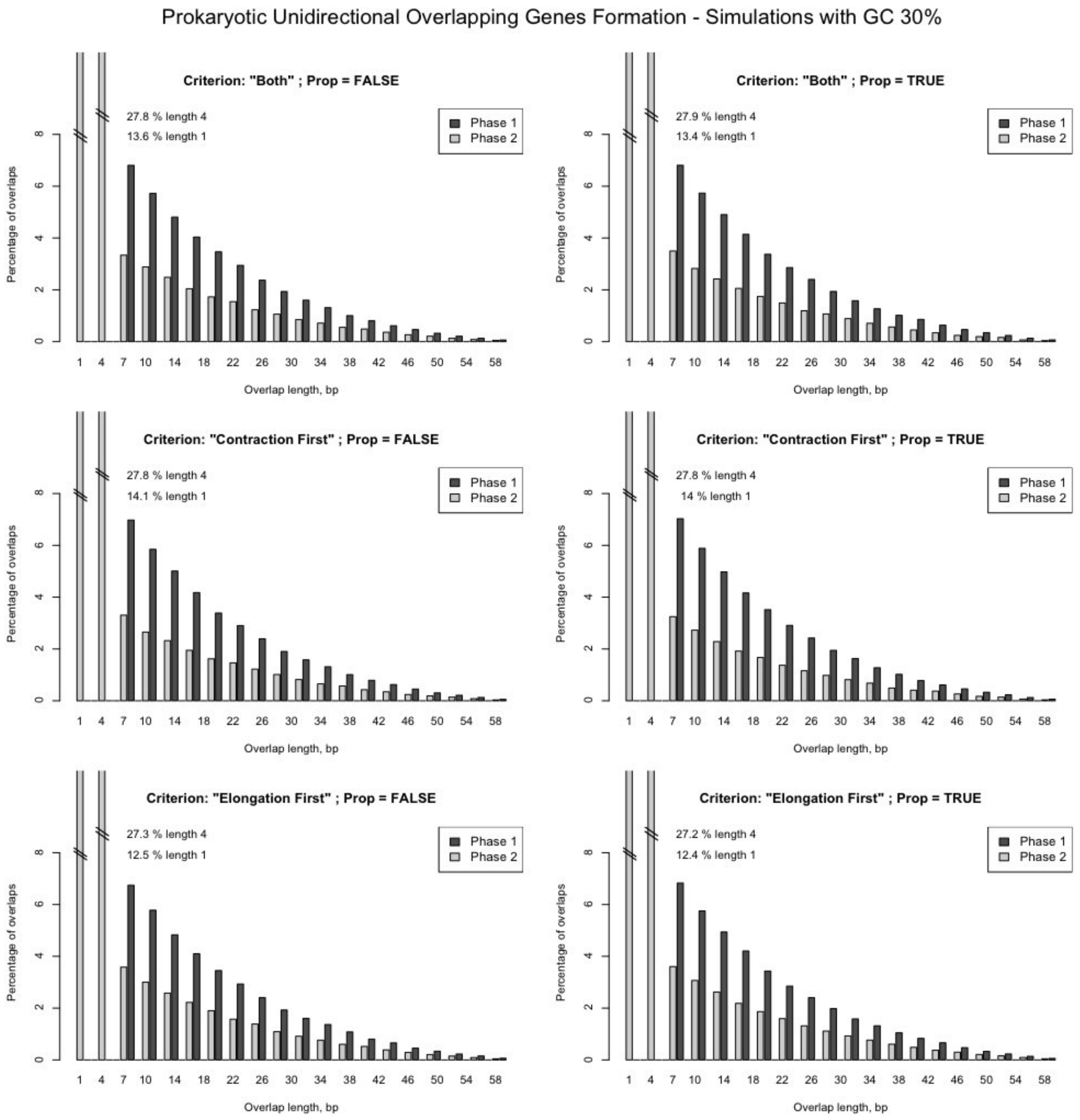

Figure S7 Hypothetical prokaryotic overlap lengths of unidirectional adjacent genes, calculated from simulated dataset (scenario 1). First set of simulations where gene size and intergenic distances were set to 63 bp and 60 bp + phase, respectively. Parameters: GC content = 30%; and all possible combinations between *criterion* (“Elongation First”, “Both”, “Contraction First”) and *Proportions of start codons* (TRUE or FALSE). Frequency of overlaps in both phases was weighted according to the mutation rate of phase 1 and phase 2 simulations (see Material and Methods).
