# Supplementary material for: Origin and Length Distribution of Unidirectional Prokaryotic Overlapping Genes
Source: G3 (Bethesda). 2013 Nov 5;4(1):19–27. doi: 10.1534/g3.113.005652 (PMC3887535; doi:10.1534/g3.113.005652)
Supplement: Supporting Information [file supp_g3.113.005652_FigureS12.pdf]

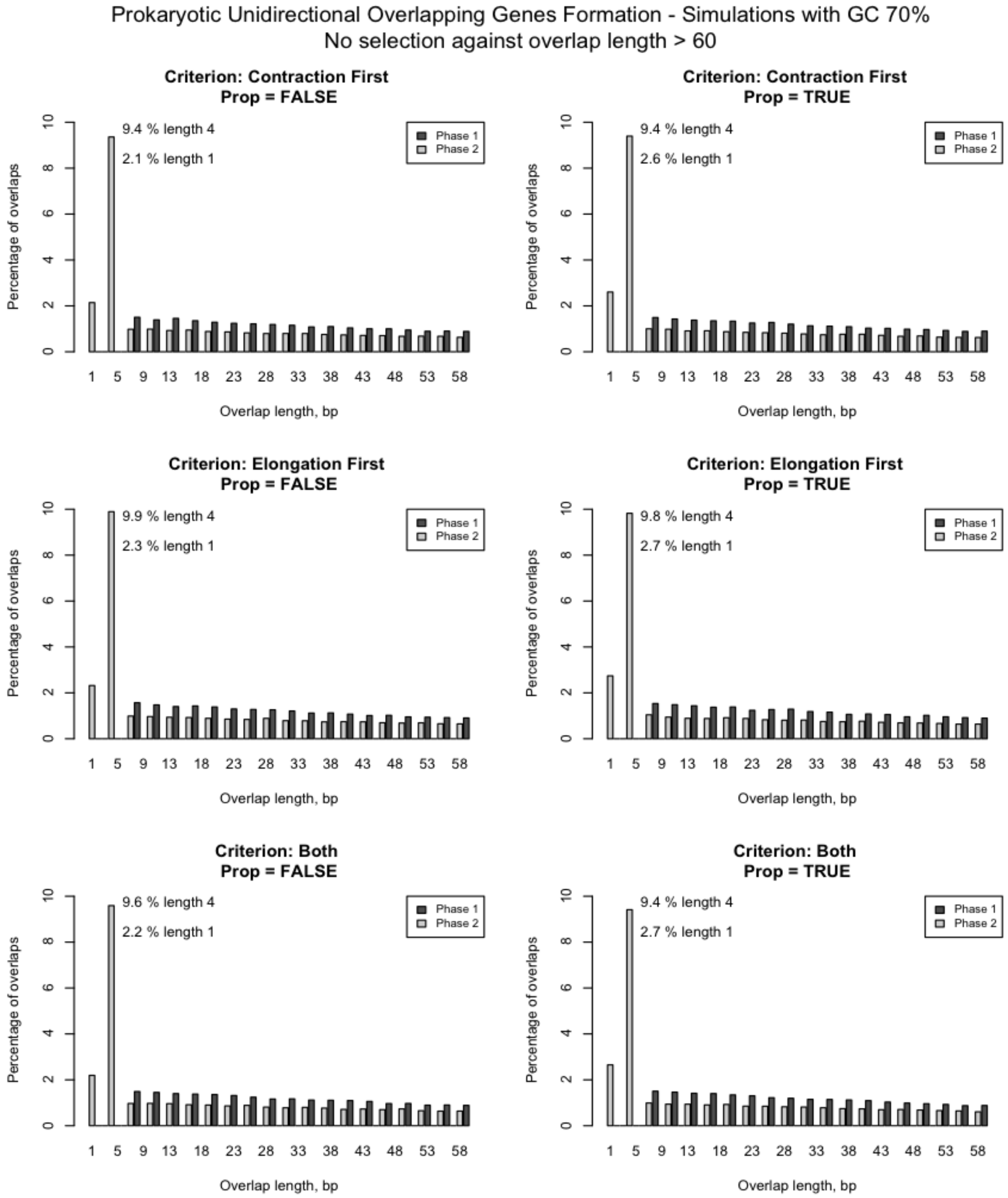

Figure S12 Hypothetical prokaryotic overlap lengths of unidirectional adjacent genes, calculated from simulated dataset (scenario 2). Second set of simulations where gene size and intergenic distances were retrieved from an empirical distribution of prokaryotic genomes (see Figures S1-S4). Parameters: GC content = 70%; and all possible combinations between *criterion* (“Elongation First”, “Both”, “Contraction First”) and *Proportions of start codons* (TRUE or FALSE). No weighting scheme was applied to the representativeness of phase 1 or phase 2. No selection against overlap length > 60 bp was included. Barplot is limited to show only overlap length < 60 bp.
