# Supplementary material for: Origin and Length Distribution of Unidirectional Prokaryotic Overlapping Genes
Source: G3 (Bethesda). 2013 Nov 5;4(1):19–27. doi: 10.1534/g3.113.005652 (PMC3887535; doi:10.1534/g3.113.005652)
Supplement: Supporting Information [file supp_g3.113.005652_FigureS16.pdf]

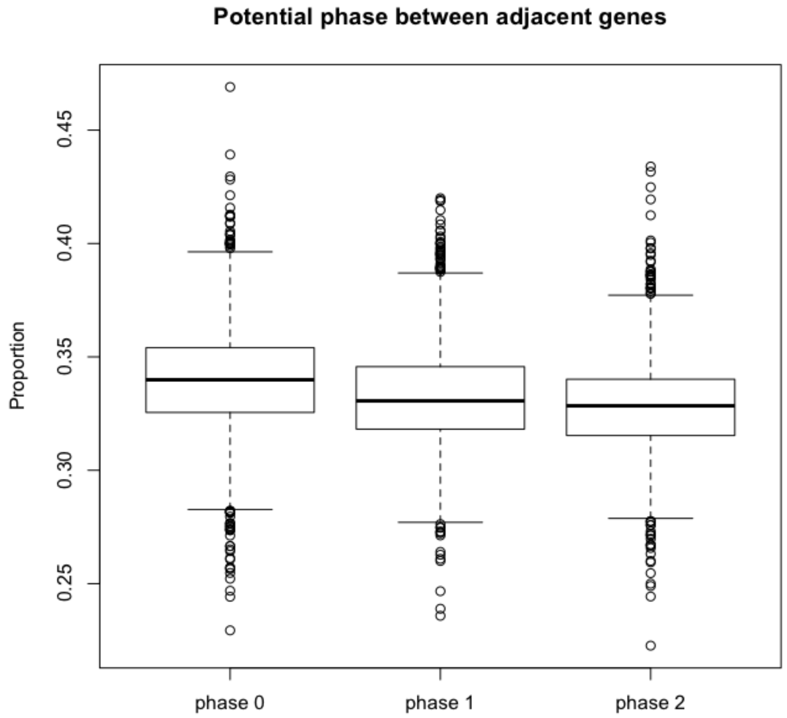

Figure S16 Potential overlapping phase between adjacent non-overlapping gene pairs. We measured the potential overlapping phase between neighboring non-overlapping genes separated by 200 bp or less. The proportions of each phase is near 1/3, although phase 0 > phase 1 > phase2.
