# Supplementary material for: Origin and Length Distribution of Unidirectional Prokaryotic Overlapping Genes
Source: G3 (Bethesda). 2013 Nov 5;4(1):19–27. doi: 10.1534/g3.113.005652 (PMC3887535; doi:10.1534/g3.113.005652)
Supplement: Supporting Information [file supp_g3.113.005652_FigureS17.pdf]

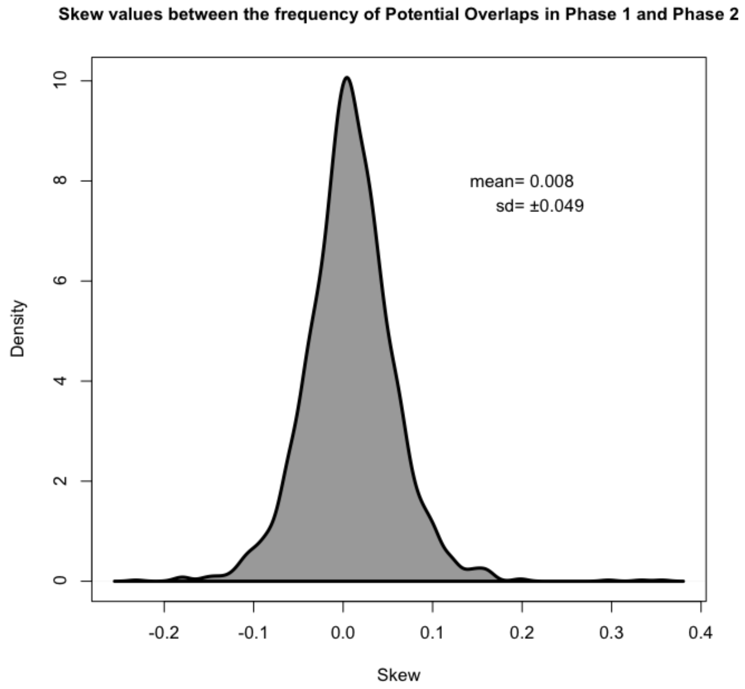

Figure S17 Distribution of the skew values for the pairwise differences between potential phase 1 and potential phase 2 overlaps. Skew values were calculated for each genome as followed: *(fpp1 – fpp2)/(fpp1 + fpp2)*, where *fpp1*: frequency of potential phase 1 overlaps and *fpp2*: frequency of potential phase 1 overlaps. Skew values can vary between -1 (no potential phase 2 overlaps) and 1 (no potential phase 1 overlaps). If skew equals to zero, then no biased distribution is found. Our results show a small mean skewed distribution towards potential phase 1 overlaps (mean = 0.008, sd = ±0.049).
