# Supplementary material for: Origin and Length Distribution of Unidirectional Prokaryotic Overlapping Genes
Source: G3 (Bethesda). 2013 Nov 5;4(1):19–27. doi: 10.1534/g3.113.005652 (PMC3887535; doi:10.1534/g3.113.005652)
Supplement: Supporting Information [file supp_g3.113.005652_FigureS18.pdf]

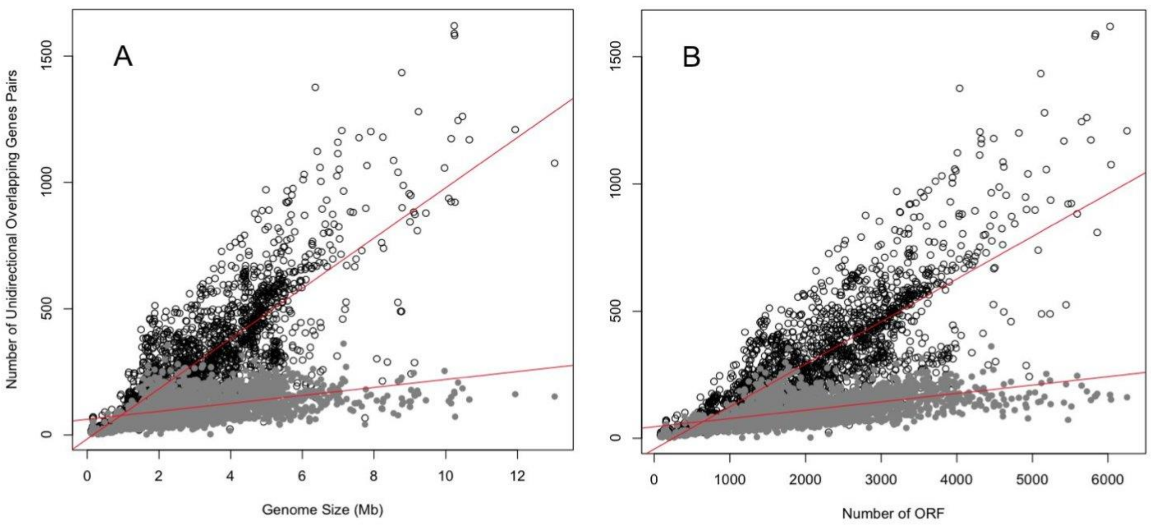

Figure S18 (A) Relationship between genome length and number of unidirectional overlapping genes pairs. Each point represents an individual genome. Closed and open circles correspond to overlapping genes pairs in phase 1 and phase 2, respectively. Linear regression (phase 1), $r^2 = 0.23$, p-value < 0.001. Linear regression (phase 2), $r^2 = 0.64$, p-value < 0.001.
(B) Relationship between total number of unidirectional pairs of open reading frames (ORF) and the number of unidirectional overlapping genes pairs. Each point represents an individual genome. Closed and open circles correspond to overlapping genes pairs in phase 1 and phase 2, respectively. Linear regression (phase 1), $r^2 = 0.35$, p-value < 0.001. Linear regression (phase 2), $r^2 = 0.63$, p-value < 0.001.

It was shown previously that the total number of ORF increases linearly with prokaryotic genome size (Mira *et al.* 2001, Fukuda *et al.* 2003). The same relationship was reported for the number of overlapping genes pairs and the genome size (Fukuda *et al.* 2003). Here, using 2,151 prokaryotic genomes, we show that these correlations are also recovered, when unidirectional overlapping genes pairs are separated into phase 1 and 2 (Figure S18 A). Additionally, we show a linear correlation of the total number of unidirectional neighboring genes pairs (potential but not overlapping genes, intergenic distance up to 100 nucleotides) and the total number of unidirectional overlapping genes (Figure S18 B). These results corroborate the hypothesis that both phase 1 and phase 2 overlapping genes have a uniform formation rate across species (Fukuda *et al.* 2003). However, it should be highlighted that prokaryotic genomes are not independent from each other, as they share an evolutionary history, and hence, these results should be analyzed with caution.
