# Supplementary material for: Origin and Length Distribution of Unidirectional Prokaryotic Overlapping Genes
Source: G3 (Bethesda). 2013 Nov 5;4(1):19–27. doi: 10.1534/g3.113.005652 (PMC3887535; doi:10.1534/g3.113.005652)
Supplement: Supporting Information [file supp_g3.113.005652_FigureS19.pdf]

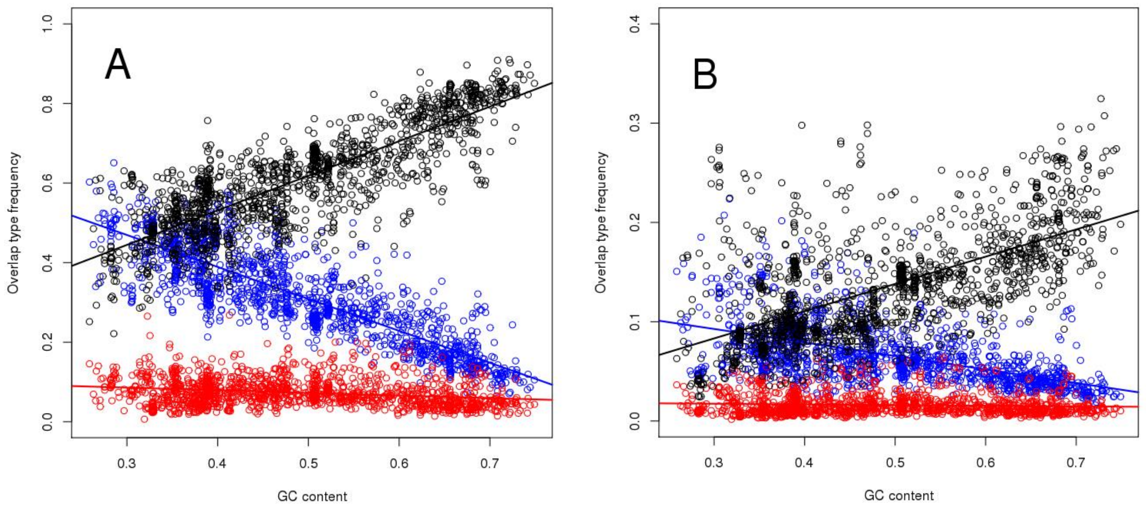

Figure S19 Relative frequency of overlapping genes in 1453 prokaryotic genomes plotted against genomic GC content. Short phase 2 overlaps are plotted in black, long phase 1 overlaps in blue and long phase-2 overlaps are plotted in red. The frequency of short phase 2 overlaps is positively correlated with genomic GC content ((A), $r^2 = 0.69$, $p < 0.001$; (B), $r^2 = 0.38$, $p < 0.001$). In contrast, the correlation between the frequency of long phase 1 and phase 2 overlaps and GC content is significantly negative (long phase 1: (A), $r^2 = 0.72$, $p < 0.001$ and (B), $r^2 = 0.31$, $p < 0.001$; long phase 2: (A) $r^2 = 0.06$, $p < 0.001$). Overlap percentages refer to the proportion of each overlap type (short phase 2, long phase 1 or long phase 2) (A) among all real overlapping genes or (B) among all real and potential overlapping genes pairs. Only genomes with 100 or more overlapping genes were considered in this analysis.

REFERENCES

Fukuda Y., Nakayama Y., Tomita M., 2003 On dynamics of overlapping genes in bacterial genomes. *Gene* **323**: 181–187.

Mira A., Ochman H., Moran M. A., 2001 Deletional bias and the evolution of bacterial genomes. *Trends Genet.* **17**: 589–596.
