# Supplementary material for: Origin and Length Distribution of Unidirectional Prokaryotic Overlapping Genes
Source: G3 (Bethesda). 2013 Nov 5;4(1):19–27. doi: 10.1534/g3.113.005652 (PMC3887535; doi:10.1534/g3.113.005652)
Supplement: Supporting Information [file supp_g3.113.005652_TableS1.pdf]

Table S1 Proportions of prokaryotic unidirectional overlapping genes in phase 1 and phase 2. Overall proportions of phase 1 and phase 2 overlapping genes pairs (OGP) are shown on the left side of the table and proportions of long phase 1 and phase 2 OGP on the right side. All taxon specific OGP were filtered from the non-redundant OGP database. Only unique overlap lengths for each homologous OGP were used in the comparison between long phase 1 and phase 2 overlaps (superscript a).

Taxonomic group	Phase 1 (%)	Phase 2 (%)	Number of overlapping genes pairs	Phase 1 long (%)	Phase 2 long (%)	Number of overlapping genes pairs[a]
Archaea						14,243
Crenarchaeota	37.5***	62.5	8,863	73.2***	26.8	5,749
Euryarchaeota	31.7***	68.3	16,696	77.6***	22.4	8,190
Korarchaeota	37.1***	62.9	321	88.3***	11.7	137
Nanoarchaeota	35.7***	64.3	143	71.8***	28.2	71
Thaumarchaeota	19.2***	80.8	442	88.5***	11.5	96
Bacteria						155,633
Acidobacteria	21.1***	78.9	4,315	78.9***	21.1	1,192
Actinobacteria	16.6***	83.4	61,133	71.9***	28.1	15,989
Aquificae	36.4***	63.6	4,141	78.5***	21.5	2,081
Bacteroidetes	36.3***	63.7	17,541	81.9***	18.1	8,743
Chlamydiae	37.1***	62.9	1,950	70.7***	29.3	1,229
Chlorobi	27.8***	72.2	2,097	76.7***	23.3	853
Chloroflexi	27.4***	72.6	4,862	81.4***	18.6	1,773
Chrysiogenetes	26.3***	73.7	590	81.6***	18.4	190
Cyanobacteria	32.1***	67.9	7,039	75.3***	24.7	3,294
Deferribacteres	45.6***	54.4	2,413	86.4***	13.6	1,427
Deinococcus-Thermus	22.5***	77.5	5,574	73.5***	26.5	1,927
Dictyoglomi	39.3***	60.7	708	79.8***	20.2	372
Elusimicrobia	32.7***	67.3	376	79.9***	20.1	154
Fibrobacteres	29.1***	70.9	461	81.0***	19.0	174
Firmicutes	43.6***	56.4	50,413	83.0***	17.0	35,673
Fusobacteria	44.4***	55.6	1,411	86.4***	13.6	778
Gemmatimonadetes	14.7***	85.3	774	84.4***	15.6	135
Nitrospirae	27.1***	72.9	1,825	75.3***	24.7	665
Planctomycetes	21.7***	78.3	3,071	64.4***	35.6	1,047
Proteobacteria	27.6***	72.4	154,188	76.3***	23.7	68,774
Spirochaetes	33.5***	66.5	9,725	82.0***	18.0	4,469
Synergistetes	37.6***	62.4	1,520	78.3***	21.7	812
Tenericutes	45.6	54.4	507	91.1***	8.9	257

Thermobaculum	40.8***	59.2	463	84.9***	15.1	225
Thermotogae	35.7***	64.3	4,908	78.8***	21.2	2,870
Verrucomicrobia	27.7***	72.3	1,439	75.3***	24.7	530

****p* value ≤ 0.001
